# MGP promotes CD8^+^ T cell exhaustion by activating the NF-κB pathway leading to liver metastasis of colorectal cancer

**DOI:** 10.7150/ijbs.70137

**Published:** 2022-03-06

**Authors:** Dawei Rong, Guangshun Sun, Zhiying Zheng, Li Liu, Xiaoyuan Chen, Fan Wu, Yichao Gu, Yongjiu Dai, Weizhe Zhong, Xiaopei Hao, Chuanyong Zhang, Xiongxiong Pan, Jinhai Tang, Weiwei Tang, Xuehao Wang

**Affiliations:** 1 School of Medicine, Southeast University, Nanjing 210000, Jiangsu, P. R. China.; 2 Hepatobiliary/Liver Transplantation Center, the First Affiliated Hospital of Nanjing Medical University, Key Laboratory of Living Donor Transplantation, Chinese Academy of Medical Sciences, Nanjing 210000, Jiangsu, P. R. China.; 3 Department of General Surgery, Nanjing First Hospital, Nanjing Medical University, Nanjing 210000 Jiangsu, P. R. China.; 4 Department of Anesthesiology, the First Affiliated Hospital of Nanjing Medical University, Nanjing 210000, Jiangsu, P. R. China.; 5 First Teaching Hospital of Tianjin University of Traditional Chinese Medicine, Tianjin 301617, P. R. China.; 6 State Key Laboratory of Modern Chinese Medicine, Tianjin University of Traditional Chinese Medicine, Tianjin 301617, P. R. China.; 7 Department of General Surgery, The First Affiliated Hospital of Nanjing Medical University, Nanjing 210000, Jiangsu, P. R. China.

**Keywords:** MGP, colorectal cancer, immune escape, liver metastasis, PD-L1

## Abstract

Matrix Gla protein (MGP) was originally reported as a physiological suppressor of ectopia calcification and has also been reported to be associated with cancer. However, the relation between the biological functions of MGP and the immune response in colorectal cancer (CRC) remains unclear. Here, we investigated the regulatory role of MGP in the immune microenvironment of CRC. MGP expression in CRC samples was assessed by single-cell RNA sequencing and the Gene Expression Omnibus (GEO) database, and confirmed by quantitative real-time Polymerase Chain Reaction (qRT-PCR) and immunohistochemistry analysis of human CRC samples. The effect of MGP on proliferation and invasion of CRC cells was evaluated by *in vitro* assays involving MGP knockdown and overexpression. Luciferase reporter assay and chromatin immunoprecipitation (ChIP)-qPCR assay were performed to identify transcriptional regulatory sites of the nuclear factor kappa-B (NF-κB) and programmed cell death ligand 1 (PD-L1). *In vivo* experiments were performed in mouse model of CRC liver metastasis established *via* spleen injection. The results revealed that MGP was significantly upregulated in cancer cell clusters from the primary CRC or liver metastases, compared with that in the corresponding paracancerous tissues via single-cell RNA sequencing. MGP enriched intracellular free Ca^2+^ levels and promoted NF-κB phosphorylation, thereby activated PD-L1 expression to promote CD8^+^ T cell exhaustion in CRC. The luciferase reporter assay and ChIP-qPCR assay indicated that the transcriptional regulation of NF-κB upregulated PD-L1 expression. *In vivo*, MGP inhibition significantly decreased the rate of CRC liver metastasis, which was further reduced after combined therapy with αPD1 (anti-PD1). In conclusions, this study revealed that MGP can facilitate CD8^+^ T cell exhaustion by activating the NF-κB pathway, leading to liver metastasis of CRC. The combination of MGP knockdown and αPD1 can synergistically resist liver metastasis of CRC.

## Introduction

Colorectal cancer (CRC) is the third leading cause of cancer-related deaths worldwide 1,2. CRC incidence and mortality rates are increasing over time, and the global burden of CRC has been predicted to increase by 60% to more than 1.1 million deaths and 2.2 million novel cases by 2030 3, 4. Metastasis is the major cause of CRC fatality. Liver metastasis has been reported as the leading cause of death in patients with CRC. As reported in previous studies, nearly 25% of CRC cases are clinically diagnosed with liver metastasis during initial stage, and approximately 50% of CRC carriers develop liver metastasis symptoms during the course of the disease. Approximately 30-50% of patients with CRC develop recurrent liver metastases after radical resection, and more than 50% among them succumb to the disease 5. Lungs are the second most common site of distant metastases in CRC 6, 7. Existing research has shown that 10-15% of patients with CRC develop lung metastases during the disease 8, 9. Compared with colon cancer, patients with rectal cancer are at a high risk of developing synchronous and atypical pulmonary metastases, which is considered to result from the direct spread of rectal cancer *via* hemorrhoid vein into the systemic circulation 8-10. However, the existing therapeutic strategies for patients with distant metastatic CRC (for example, surgery, radiotherapy, and chemotherapy) remain unsatisfactory for rapid CRC metastasis deterioration and against drug resistance.

Cancer immunotherapy has attracted unprecedented attention due to clinical success of checkpoint blockade immunotherapy in several types of cancer 11,12. Programmed cell death protein 1/programmed death ligand 1 (PD1/PD-L1) blockade therapy was found effective in microsatellite instability-high and mismatch repair (MMR)-deficient CRC, a type of CRC exhibiting high somatic mutations; however the type is represented by only 5% of the patient population 13. Therefore, the cause of PD1/PD-L1 activation and the mechanisms leading to immune escape should be elucidated. This study explored the effect of matrix Gla protein (MGP), an extracellular matrix (ECM) protein, on CRC immunotherapy responses. MGP has been recognized as a secreted, calcium-binding matrix protein, which comprises 5-6 post-translationally modified γ-carboxyglutamic acid residues obtained *via* vitamin K-dependent carboxylation 14. It is synthesized in cartilage, bone, and other tissues (for example, lung, heart, kidney, and vascular smooth muscle cells) 15, 16. Extensive studies have been conducted on the function of MGP in the ECM 17, 18, but the relation between intracellular MGP and immunity remains unclear. We investigated the effect of MGP on CRC liver metastasis and further revealed that MGP could facilitate CD8^+^ T cell exhaustion by activating the nuclear factor kappa-B (NF-κB) pathway. The combination of MGP knockdown and αPD1 (anti-PD1) contributed to synergistic resistance against liver metastasis, which may provide new insights into CRC immunotherapy.

## Materials and methods

### Cells and cell culture

The human colonic normal epithelial cell line NCM460, human CRC cell lines (HT29, HCT116, SW480, CACO2, and HCT8), and mouse CRC cell lines (MC38 and CT26) were obtained from the Cell Bank of Type Culture Collection (Chinese Academy of Sciences, China). Cells were cultured in Dulbecco's modified Eagle's medium (DMEM; Gibco,USA) supplemented with 10% fetal bovine serum (FBS; Gibco, USA). The cell lines were maintained at 37 °C in a constant‐temperature incubator with 5% CO_2_.

### Animal model

The animal experiment was approved by the Animal Management Committee of Nanjing Medical University, and all experimental procedures and animal care followed the institutional ethics guidelines for animal-related experiments. All mice were bred under specific pathogen-free (SPF) conditions at the Nanjing Medical University Laboratory Animal Center. The mice were euthanized by cervical dislocation.

To establish subcutaneous tumor-bearing mouse model, MC38 cells or CT26 cells (1 × 10^6^) treated with MGP shRNA (sh-MGP) were inoculated into the right groin of C57BL/6 or BALB/C mice (*n* = 5 per group), respectively. Cells treated with control shRNA (sh-NC) were used as the control. The tumor size was measured every 4 days and the tumor volume was calculated using the following formula: volume (mm^3^) = width^2^ × length/2. On day 24, the mice were sacrificed, and their livers were examined for protein expression using immunohistochemistry analysis.

CRC liver metastasis mouse model: MC38 cells or CT26 cells (1 × 10^6^) were transplanted into the spleen of male C57BL/6 or BALB/C mice aged five weeks, respectively. The specific procedure for spleen transplantation is presented below. The mice were weighed and anesthetized with 0.5% pentobarbital sodium (50 mg/kg) *via* intraperitoneal injection. The mice were then fixed on the operating table with adhesive tape for nearly 3-5 min. After disinfecting the aneroid skin, a longitudinal incision (approximately 0.5 cm) was made under the posterior costal margin of the left axillary line of the mouse. A willow leaf spleen found in the abdomen was pulled out of the abdominal cavity. Subsequently, 0.2 mL of the single-cell suspension was injected into the lower pole of the spleen, and an alcohol swab was pressed on the injection site. After no bleeding was detected, the muscle and skin of the abdomen were closed with intermittent suturing. After the operation, the mice were maintained under SPF condition in an animal laboratory. Different groups were as follows: sh-NC, sh-MGP, sh-MGP + phosphate-buffered saline (PBS), and sh-MGP + αPD1 (BP0273; Bioxcell,USA) (*n* = 5 for each group). In the sh-MGP + αPD1 group, 6.6 mg/kg intraperitoneal injection of αPD1 was administered on the first day, and once every three days thereafter. On day 19, the mice were sacrificed, and their livers were examined for protein expression using immunohistochemistry analysis.

### Patients and tissue specimen collection

In accordance with the Declaration of Helsinki, all participants were informed about this study, and written informed consent was obtained from all patients before the study was conducted. The primary CRC and corresponding intestinal tissue, liver metastasis cancer tissue and corresponding normal liver tissue, and preoperative blood were collected from a patient with the primary CRC to conduct single-cell RNA sequencing. Fresh cancer tissue and corresponding intestinal tissue samples were collected from five patients with CRC for quantitative reverse transcription polymerase chain reaction (qRT-PCR) and western blotting. Moreover, 57 samples of CRC tissue and the adjacent paracancerous tissue stored in liquid nitrogen were obtained from random patients admitted at the First Affiliated Hospital of Nanjing Medical University and Nanjing First Hospital. The collection of human specimens was approved by the Medical Ethics Committee of Nanjing Medical University. The overall samples removed from the body were efficiently collected. The samples were immediately frozen in liquid nitrogen and stored at -80°C until further use. Experienced clinicians analyzed and classified the collected tumor specimens. Next, 57 CRC patient's clinical information, including age, gender, stage, tumor size, differentiation, tumor lymph node metastasis stage,and lymph node metastasis were collected. A regular follow-up was performed for all patients; the overall survival (OS) period ranged from the date of surgery to the date of death or the last follow-up visit.

### Single-cell RNA sequencing analysis

The primary CRC and corresponding intestinal tissue, and liver metastasis cancer tissue and corresponding liver tissue were collected after surgical resection and stored in DMEM, high glucose, GlutaMAX™ Supplement, HEPES (Thermo Fisher, USA) in 1% bovine serum albumin (BSA; Thermo Fisher, USA) in a 15 mL conical tube and transported on ice to the laboratory. Subsequently, we transferred the tissue samples to a 35 × 12 mm^2^ Petri dish (Thermo Fisher, USA) and minced them to 0.5-1.0 mm^2^ fragments using a sterile surgical scalpel in approximately 1 mL of the media. Tissue digestion was performed using Liberase™ TH Research Grade (Sigma-Aldrich, USA) and Accutase® solution (Sigma-Aldrich, USA). Single cells were filtered for downstream analysis according to the following criteria: the unique molecular identifier (UMI) count was between 3,000 and 40,000, and the mitochondrial percentage was less than ten percent of the total UMI count. Gene expression (in UMI) was scale-normalized and transformed into log2 (UMI+1). Raw counts were generated using Illumina Hiseq4000 sequencer (Singleron Botechnologies, China) using the 10× Genomics Cell Ranger pipeline (version 2.1.0; 10× Genomics, Delaware, USA). The primary CRC tissues (cell number, *n* = 4688) and corresponding intestinal tissues (cell number, *n* = 4043), liver metastasis cancer tissues (cell number, *n* = 7813) and corresponding liver tissues (cell number, *n* = 5956), and preoperative blood (cell number, *n* = 4855) were analyzed.

### RNA extraction and qRT-PCR

Total RNA was isolated from tissues and cells using TRIzol reagent (Invitrogen,USA) according to the manufacturer's protocol. Using reverse transcription kit (Takara, Japan), we reverse transcribed RNA into cDNA. All primer sequences are listed in Table S1. The internal control (GAPDH) was used to normalize mRNA expression levels.

### Cell transfection

We constructed plasmids for MGP and lentivirus packaging (Genechem, China) in human CRC cells (HCT116 and HT29). MGP was downregulated in both human CRC cells (HCT116 and HT29) and mouse CRC cells (MC38 and CT26) via shRNA (Genechem, China). Polybrene (Sigma-Aldrich, USA) at a concentration of 6 µg/mL and an appropriate amount of virus was added to 2 × 10^5^ cells/mL, and mixed thoroughly. After incubation at 37 °C for 4 h, the same amount of fresh medium was added to dilute polybrene. In particular, for the co-transfected cell lines (sh-MGP+sh-PD-L1), we first constructed the sh3-MGP stable transfected cell line, after 14 days of screening with puromycin, the above transfection procedure was repeated using sh-PD-L1 lentivirus, followed by secondary screening with puromycin, and finally co-transfected cell lines were obtained. Table S1 lists the sequences of shRNAs used in this study. sh-PD-L1 and sh-p65/si-p65 were obtained from Genechem Biotechnology (Genechem, China). Transfection efficiency was determined by qRT-PCR and western blotting.

### Cell proliferation assay

In the clone forming experiment, CRC cells were seeded in 6-well plates at a density of 1000 cells/well. After 10 days, the cells were fixed using methanol (4%; Wobixin Inc., China), followed by staining with Giemsa (Sigma-Aldrich, USA), and the colonies were then imaged and counted.

To perform the CCK-8 assay, we seeded 10^3^ CRC cells in 96-well plates and then treated them with 10 μL of CCK-8 solution (RiboBio, China) at 0 h, 24 h, 48 h, 72 h, and 96 h of culturing. The cell absorbance was measured at the respective time points at 450 nm using a microplate reading element, according to the manufacturer's instructions (Synergy, USA).

Using Cell-Light 5-ethynyl-2'-deoxyuridine (EdU) DNA Cell Proliferation Kit (RiboBio, China), we performed the EdU experiment to assess cell proliferation. We plated 5 × 10^4^ CRC cells in 24-well plates, and the cells were then cultured for 24 h. The cell lines were fixed with 4% paraformaldehyde after incubating them with 50 mmol/L EdU solution for 2 h. Following the manufacturer's protocol, we treated the cell lines with Apollo Dye Solution and Hoechst seal, respectively. EdU cell lines were captured and counted under Olympus FSX100 microscope (Olympus, Japan).

### Cell apoptosis assay

CRC cells were stained with Annexin V-PE and 7-aminoactinomycin D (7-AAD) using an apoptosis detection kit (BD Biosciences, USA). We examined the stained cells using FACScan (BD Biosciences, USA). Furthermore, we studied the apoptosis of various cell lines using FlowJo V10 software (Tree Star, USA).

### Transwell invasion assay

We seeded 10^4^ CRC cells in 200 μL serum-free medium in the upper chamber, according to the manufacturer's protocol. The matrix mixture was placed in the Transwell chamber (Corning, USA) for the invasion test. The bottom cavity was filled with the culture medium and 10% FBS as a chemical attractant for cancer cells. After incubation for 24 h, cells in the upper compartment were fixed with methanol (4%; Wobixin Inc., China), and then stained with crystal violet (Wobixin Inc., China) for 15 min. The cells were visualized by imaging, and cell counting was performed.

### Immunofluorescence and immunohistochemistry

We sectioned paraffin-embedded samples to 4-mm thickness. Antigen retrieval was performed by pressure cooking for 3 min in 0.01 mol/L citrate buffer (pH 6.0; Wobixin Inc., China). Immunofluorescence cells were fixed with 4% paraformaldehyde (Wobixin Inc., China) for 20 min at ambient temperature and permeabilized with 0.05% Triton X-100 (Sigma-Aldrich, USA) in PBS for 5 min. Samples were blocked in PBS with 2% BSA for 1 h at ambient temperature and incubated overnight with antibodies specific for MGP (1:200; Abcam,UK) and PD-L1(1:200; Abcam, UK) at 4 °C, and subsequently with Alexa Fluor or HRP-conjugated secondary antibodies (Abcam, UK) for 1 h at ambient temperature. Hoechst 33342 (Sigma-Aldrich, USA) was used to counterstain the nuclei, and images were obtained using laser scanning confocal microscopy (Zeiss, Germany).

For immunohistochemistry, specimens were incubated overnight with antibodies specific for MGP (1:200; Abcam, UK), CD8 (1:200; Abcam, UK), PD-L1 (1:200; Abcam, UK), and Ki-67 (1:200; Abcam, UK) at 4 °C, and immunodetection was performed the following day using 3'-diaminobenzidine (DAB; Wobixin Inc., China), according to the manufacturer's instructions.

### Preparation of antigen-specific CD8^+^ T cells

Acquisition of dendritic cells: Peripheral blood mononuclear cells (PBMCs) were isolated from the blood samples of patients with CRC and cultured in RPMI 1640 containing 1% serum for 1 h. IL-4 (50 ng/mL; PeproTech, USA) and granulocyte-macrophage colony-stimulating factor (GM-CSF, 100 ng/mL; PeproTech, USA) were added to PBMCs and cultured for seven days. Subsequently, heat-stimulated human CRC cells (grouped as follows: sh-NC, sh-MGP, vector, MGP) were added and co-cultured for one day. We thus obtained CRC antigen-loaded antigen-presenting cells.

Initial CD8^+^ T cells were separated from PBMCs using magnetic beads, and 1 μg/mL CD3 mAb (Miltenyi Biotec, Germany), 5 μg/mL CD28 mAb (Miltenyi Biotec, Germany), 20 ng/mL recombinant human interleukin-2 (IL-2; PeproTech, USA), and 50 U/mL penicillin (Wobixin Inc., China) were added to the initial CD8^+^ T cells. Antigen-responsive CD8^+^ T cells were obtained by co-culturing CRC antigen-loaded antigen-presenting cells with initial CD8^+^ T cells for three days. Finally, antigen-specific CD8^+^ T cells were obtained by co-culturing antigen-responsive CD8^+^ T cells with CRC cells for 24 h.

### Western blotting

Proteins were extracted from the cell using RIPA buffer (Sigma-Aldrich, USA), resolved by SDS-polyacrylamide gel, and then transferred to PVDF membranes (Wobixin Inc., China). The primary antibodies (Abcam, UK) against MGP, PD-L1, cAMP-response element binding protein (CREB), p-CREB, nuclear factor of activated T-cells (NFATC1), p-NFATC1, P65, p-P65, c-MYC, and COX-2 were used. Peroxidase-conjugated secondary antibody (CST, Sigma-Aldrich,USA) was used, and the antigen-antibody reaction was visualized by enhanced chemiluminescence assay (ECL; Thermo Fisher, USA).

### Luciferase reporter assay

The pGL3‐PD‐L1 was constructed by inserting the 2200 kb human PD‐L1 promoter (-2000 to +200 nt) into the vector of pGL3‐basic (Genechem,China). To determine the minimal PD‐L1 promoter sequence required for constitutive and inducible activity, we constructed a series of promoter deletion fragments by qRT-PCR and cloned them into the same reporter vector: -2000 to +200 nt, -1500 to +200 nt, -1000 to +200 nt, and 0 to +200 nt. The plasmids of pGL3‐PD‐L1 mutant #1 (TGGACTTTCC→TGTACGCGCC, pGL3‐PD‐L1‐M1) were generated with the above-mentioned wild-type pGL3‐PD‐L1 as the template using a site‐directed mutagenesis kit (Genechem, China), according to the manufacturer's instructions. The luciferase activity was determined using the Dual-Luciferase Reporter Assay System (Promega, USA).

### Chromatin immunoprecipitation (ChIP)-qPCR assay

We performed the ChIP assay using the EZ-Magna ChIP Chromatin Immunoprecipitation Kit (Abcam, UK), according to the manufacturer's instructions. In brief, we fixed 5 × 10^6^ CRC cells in 1% formaldehyde for 10 min at ambient temperature. The fixed cells were harvested, lysed, and sonicated for 10 cycles of 10s ON/20s OFF and 50% AMPL using Sonics VCX130 (Sonics & Materials, USA). Antibodies against p65 (Thermo Fisher, USA) and rabbit IgG (Thermo Fisher, USA) were used for immunoprecipitation. PCR amplification of the precipitated DNA was performed. Table S1 lists the primer sequences used for the ChIP assay.

### RNA sequencing and pathway analysis

Total RNA was isolated from 5 × 10^6^ CRC cells treated with sh-MGP or sh-NC. Library construction and sequencing were performed by the Beijing Novogene Corporation (Novogene, China). lncRNAs, mRNAs, and circRNAs were sequenced. The original image data files obtained by high-throughput sequencing (Illumina HiSeqTM2500, Novogene, China) were converted into sequenced reads through base calling analysis. The Kyoto Encyclopedia of Genes and Genomes (KEGG) pathway annotation and enrichment were conducted using the DAVID (https://david.ncifcrf.gov/). We considered pathways with Q value ≤ 0.05 as significantly enriched. Functional annotation was conducted by complying with the Gene Ontology database (for example, biological process, cellular component, and molecular function classifications). We performed Fisher exact test for selecting only significant categories, and the Gene Ontology (GO) terms with computed Q value ≤ 0.05 showed statistical significance.

### Fluo-3 AM staining

Fluo-3 AM (Beyotime, Shanghai, China) reserve solution (5 mmol/L) was mixed with Hanks' Balance Salt Solution (HBSS) (0.39 g/L KCl, 0.07 g/L KH_2_PO_4_, 8.06 g/L NaCl, 0.10 g/L Na_2_HPO_4_·7H_2_O, 0.24 g/L CaCl_2_, 0.10 g/L MgCl_2_, 0.10 g/L MgSO_4_, and 1.52 g/L D-glucose) to a final concentration of 5 μmol/L. The whole dyeing process of Fluo-3 AM consists of two stages of loading and de-esterification. The cells were loaded with 5 μmol/L Fluo-3 AM at 37 °C. Subsequently, the cells were washed with pure HBSS and placed for 30 min at 37 °C for de-esterification. Next, the cells were imaged under a confocal microscope (FluoView FV1200; Olympus, Japan). The field of vision was randomly selected, and the average fluorescence intensity of the cells was determined for statistical analysis. After CRC cells were loaded with Fluo-3 AM, trypsin digestion and HBSS washing were performed three times. Intracellular calcium ion concentration was measured, and the curve was drawn using the FACS software (BD Biosciences, USA).

### Mass cytometry

We obtained the liver cancer tissue samples of the respective groups (sh-NC, sh-MGP, sh-MGP+PBS, and sh-MGP+αPD1) treated with MC38 cell line. We used the Miltenyi Mouse Tumor Dissociation Kit (Miltenyi Biotec, Germany) for processing of tumor tissues from mouse, in which Percoll removes debris and splits red blood cells. Overall, 3 × 10^6^ cells were obtained. CyTOF staining consisted of the following steps: ^194^Pt staining → Fc block → surface antibody staining → overnight DNA staining (^191/193^Ir) → intracellular antibody staining → collecting data on the computer. The data analysis steps comprised FlowJo pretreatment (circle and select single, live, and complete CD45^+^ immune cells), followed by bio-information analysis (X-shift algorithm performs cell subpopulation clustering, manual annotation, TSNE dimensionality reduction visual display, and statistical analysis). This experiment was performed at the PLTTECH Company (Plttech, China).

### TCGA data analysis, GSE analysis and site prediction

TCGAportal (http://tumorsurvival.org/index.html) was used to predict the correlation between MGP expression and the prognosis of patients with CRC. The hTFtarget database (http://bioinfo.life.hust.edu.cn/hTFtarget#!/) was used to predict the transcription binding sites of NF-κB and PD-L1. The JASPAR database (http://jaspar.genereg.net/) was used to predict the p65 binding sites in the PD-L1 gene promoter. ChIP data of human NF-κB-PD-L1 binding sites was obtained from GSE131710. The correlation between MGP expression and immune factors was predicted using the TISIDB database (http://cis.hku.hk/TISIDB/index.php). The correlation between MGP expression and pathway enrichment in tumors was performed with GSE20842 and GSE6988 datasets. We downloaded the data from GSE156429 database and compared the RNA sequencing results of differential genes in primary colorectal cancer and liver metastases in mouse models.

### Statistical analysis

We carried out statistical analyses using the GraphPad Prism 8.0 (GraphPad, USA), and *a P* value < 0.05 was considered to be statistically significant. We performed independent *t-*test for comparison of continuous variables between the two groups, whereas categorical variables were compared using the chi-square test. The Kaplan-Meier approach and univariate and multivariate analyses along with the log-rank test were used to assess survival rates.

## Results

### MGP was overexpressed in CRC tissues that indicated a worse clinical prognosis

**Figure 1:**
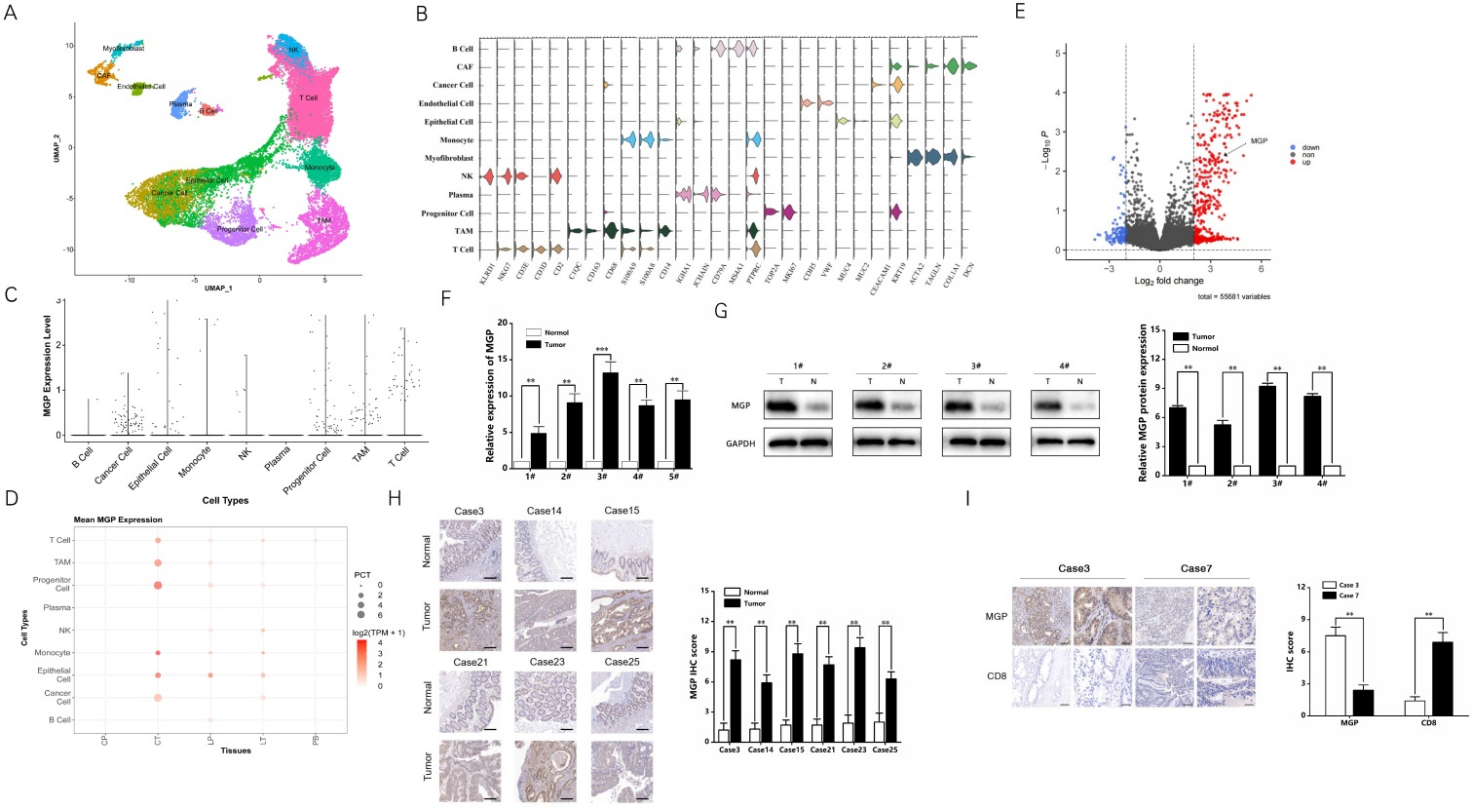
** MGP was overexpressed in CRC tissues compared with that in the adjacent colorectal tissues. (A)** UMAP plot showing clusters of all cells from CRC case. There were 12 groups defined (B cell, CAF, cancer cell, endothelial cell, epithelial cell, monocytes, myofibroblast, NK cell, plasma, progenitor cell, TAM, and T cell).**(B)** The violin plot showing marker gene expression in different cell clusters. **(C)** The bar chart showing MGP expression in different cell clusters. More number of points indicates greater number of cells. **(D)** The dot plot showing MGP expression in different cell clusters from different samples. CP, paracancerous tissue of the primary CRC; CT, primary CRC tissue; LP, paracancerous tissue of liver metastasis; LT, liver metastatic tissue; and PB, preoperative blood. **(E)** Volcanic map of differential genes of the primary and secondary foci in mouse model of CRC liver metastasis. Red color indicates higher expression in metastatic foci than that in the primary foci, whereas blue color indicates lower expression. MGP is indicated by arrows. **(F)** The mRNA expression of MGP in CRC tissues and normal tissues based on qRT-PCR. In general, five pairs (1#-5#) of tissues were tested.**(G)** The protein expression of MGP in CRC tissues and normal tissues based on western blotting. Four pairs of tissues were tested in total (1#-4#). The upper image represents the result of protein banding and the lower image illustrates the result of protein gray value analysis. **(H)** Immunohistochemistry was used to verify the protein expression of MGP in CRC tissues and paracancerous tissues collected from six cases. The left panel displays the image. The right panel represents the analysis result.**(I)** Immunohistochemistry was used to verify the protein expressions of MGP and CD8 in CRC tissues of two cases (case 3 with high MGP expression and case 7 with low MGP expression). The left panel displays the image. The right panel represents the analysis result.**, *P* < 0.01; ***, *P* < 0.001.

Single-cell RNA sequencing provides better insights into cell behavior in the context of a complex tumor microenvironment, while profiling populations of cells on an individual cell basis. In this study, the primary CRC, adjacent normal intestinal tissue, CRC liver metastasis cancer tissue, adjacent normal liver tissue, and preoperative blood samples were used for single-cell RNA sequencing. Considering the definition of classification of specific gene markers using UMAP plot (Figure 1A and 1B), we identified 12 cell clusters, which consisted of B lymphocytes (B cells), cancer associated fibroblasts (CAFs), cancer cells, endothelial cells, epithelial cells, monocytes, myofibroblasts, natural killer (NK) cells, plasma, progenitor cells, tumor-associated macrophages (TAM), and T cells. For instance, the CAF cluster specifically expresses COL1A1, DCN, and others, while endothelial cell clusters express VWF, CDH5, and others (Figure 1B). The bar chart shows that MGP was relatively highly expressed in the total sample analysis of the cancer cell population (Figure 1C). According to the dot plot, compared with samples from different parts of the body, MGP was significantly upregulated in cancer cell clusters from the primary CRC tissues; moreover, compared with paracancerous tissues, it was highly expressed in metastatic foci of CRC (Figure 1D). In addition, we compared RNA sequencing results of differential genes in the primary colorectal cancer and liver metastases in mouse models based on the data from the GSE156429 database. The results showed that MGP was expressed in both primary and metastatic tissues; however, its expression was comparatively high in metastatic tissues (Figure 1E). We performed qRT-PCR to detect MGP expression in the primary and paracancerous tissues collected from five patients with CRC, and we detected MGP protein expression in four of the cases using western blotting. According to the results, the mRNA and protein expression of MGP in CRC tissues was enhanced compared with that in paracancerous normal tissues (Figure 1F and 1G). Immunohistochemistry further indicated that MGP protein was overexpressed in CRC tissues compared with that in the adjacent paracancerous tissues (Figure 1H); a higher expression of MGP led to lower CD8^+^ T cell infiltration, and *vice versa* (Figure 1I). Furthermore, in CRC liver metastasis mouse model, MGP expression in liver metastasis tissues was significantly higher than that in normal liver tissues of eight C57BL/6 mice, as determined by western blotting (Figure S1), consistent with the results of human samples.

**Figure 2:**
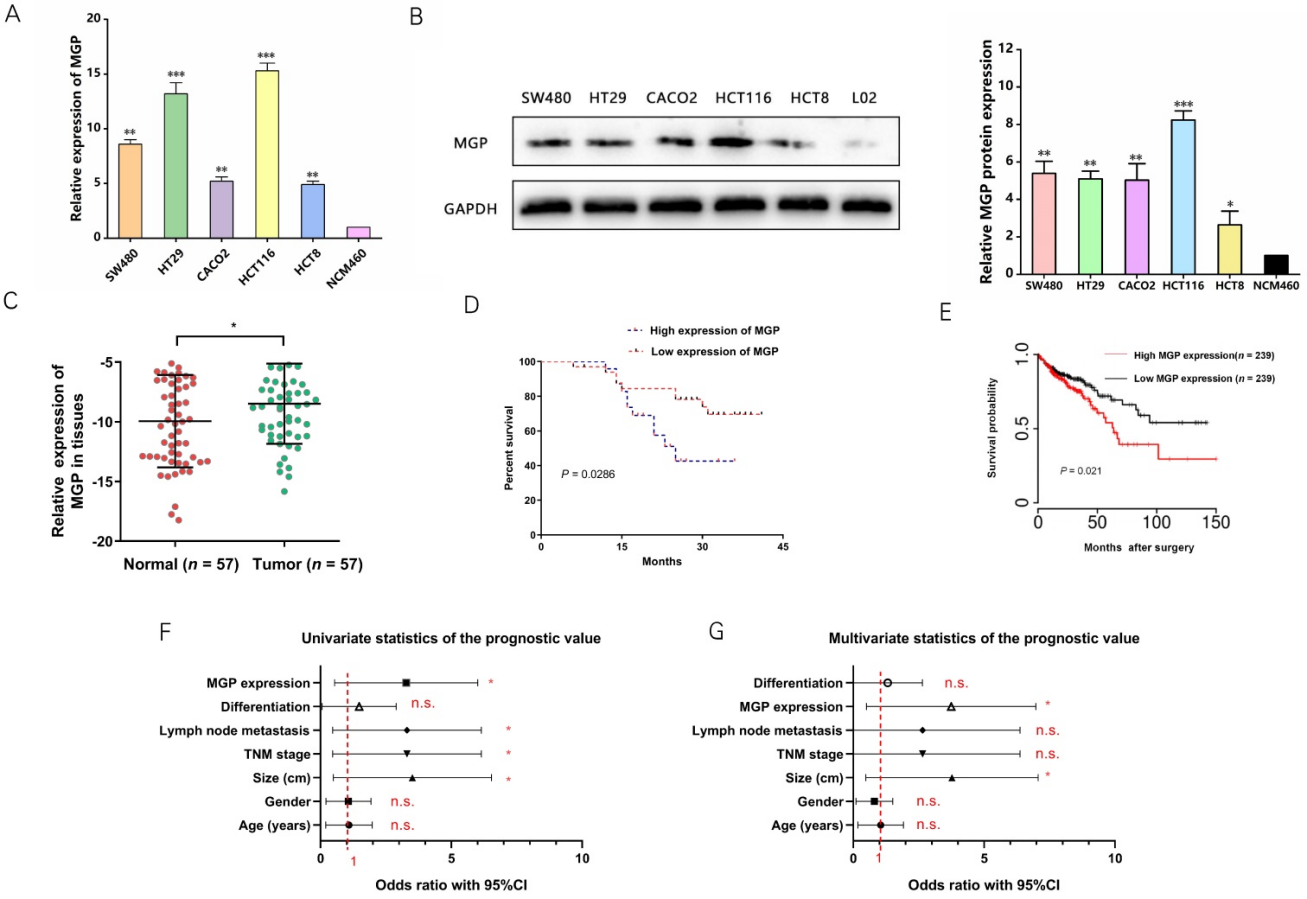
** High expression of MGP indicated a worse clinical prognosis in CRC. (A)** qRT-PCR for the abundance of MGP mRNA in CRC cells (SW480, HT29, CACO2, HCT116, and HCT8) and normal cells (NCM460). **(B)** Western blotting for the abundance of MGP protein in CRC cells and normal cells. The left panel illustrates the results of protein banding, and the right panel represents the results of protein gray value analysis. **(C)** MGP expression was measured by qRT-PCR in 57 pairs of CRC tumor tissues (Tumor) and matched noncancerous tissues (Normal). **(D)** Kaplan Meier survival curve revealed the correlation between MGP expression and overall survival. **(E)** Survival time analysis of the TCGA dataset. **(F-G)** Univariate (F) and Multivariate (G) regression analysis showing predictors of CRC prognosis.*, *P* < 0.05; **, *P* < 0.01; ***, *P* < 0.001; n.s., no significance.

qRT-PCR and western blotting were performed to measure MGP expression in human CRC cells. According to the results, MGP expression was upregulated in CRC cells (HT29, HCT116, SW480, CACO2, and HCT8) compared with that in normal intestinal cells (NCM460), with maximal expression in HCT116 and HT29 cells; therefore, HCT116 and HT29 cells were used for subsequent validation (Figure 2A and 2B). Next, MGP expression in 57 samples of human CRC tissues and adjacent normal tissues was measured by qRT-PCR, and MGP was found to be significantly overexpressed in CRC tissues compared with that in the corresponding paracancerous tissues (Figure 2C). According to the clinicopathological features, MGP overexpression was positively correlated with TNM classification and lymph node metastasis, whereas it was not significantly correlated with age, gender, tumor size, and differentiation (Table S2). According to the Kaplan-Meier survival curve, patients with CRC and high MGP expression had reduced overall survival (Figure 2D), which was consistent with the TCGA database assessment (Figure 2E). Univariate and multivariate analyses indicated high MGP expression as an independent indicator of prognosis and overall survival in patients with CRC (Figure 2F and 2G). These findings indicated that MGP was significantly upregulated in CRC and indicated poor prognosis in patients with CRC.

### MGP facilitated the proliferation and invasion of human CRC cells

**Figure 3:**
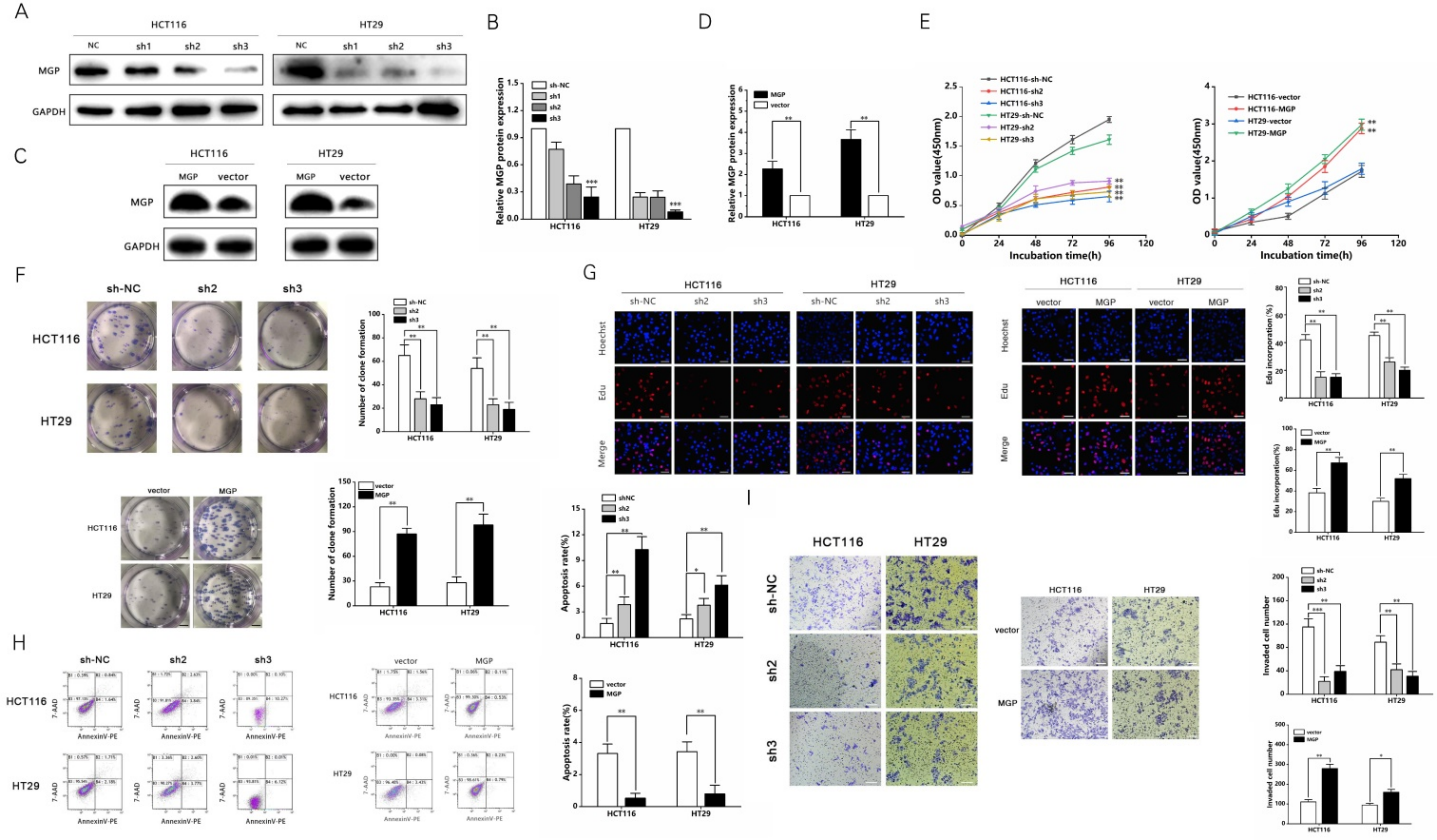
** MGP promoted the proliferation and invasion of CRC cells. (A)** Three shRNAs (sh1, sh2, and sh3) were designed to silence MGP in CRC cells (HCT116 and HT29), and validated by western blotting. **(B)** The gray value analysis results of A. **(C)** MGP overexpression was validated by western blotting in CRC cells (HCT116 and HT29). **(D)** The gray value analysis results of C. **(E)** The growth curves of cells were plotted after transfection with sh2/3-MGP/MGP based on CCK-8 assay. Left panel represents the cell proliferation result after MGP knockdown, and the right panel illustrates the cell proliferation result after MGP overexpression. **(F)** Colony formation assay was performed to assess cell proliferation. Left panel represents the cell proliferation after MGP knockdown and overexpression. Right panel displays the result of cell count analysis. **(G)** EdU assay was performed to assess cell proliferation of CRC cells transfected with sh2/3-MGP/MGP. The left panel represents the cell proliferation after MGP knockdown and overexpression. The right panel displays the result of cell count analysis. **(H)** Flow cytometry was used to assess cell apoptosis in CRC cells transfected with sh2/3-MGP/MGP. The left panel displays the cell apoptosis result after MGP knockdown and overexpression. The right panel represents the result of apoptotic cell count analysis. **(I)** Transwell invasion assay was performed to assess invasion of CRC cells transfected with sh2/3-MGP/MGP. The left panel shows the cell invasion result after MGP knockdown and overexpression. The right panel displays the result of migrated cell count analysis. *, *P* < 0.05; **,* P* < 0.01; ***,* P* < 0.001.

Three shRNAs against MGP (sh-MGP) were developed to silence MGP in HCT116 and HT29 cells. sh2-MGP and sh3-MGP were employed for evaluating cell proliferation inhibition efficiency by qRT-PCR and western blotting (Figure 3A and 3B, Figure S2A). In addition, a model of MGP overexpression was established successfully through lentivirus transfection, and inhibition efficiency was measured (Figure 3C and 3D, Figure S2B). sh2-MGP and sh3-MGP inhibited proliferation of HCT116 and HT29 cells, as per the results of CCK-8, colony formation, and EdU assays (Figure 3E-3G). Flow cytometry revealed that sh2-MGP and sh3-MGP facilitated cancer cell apoptosis (Figure 3H). Transwell invasion assay indicated that sh2-MGP and sh3-MGP significantly hindered the invasion functions of HCT116 and HT29 cells (Figure 3I). In contrast, MGP overexpression model exerted the opposite effects (Figure 3E-3I).

### MGP induced CD8^+^ T cell exhaustion when co-cultured with antigen-specific CD8^+^ T cells isolated from PBMC samples

**Figure 4:**
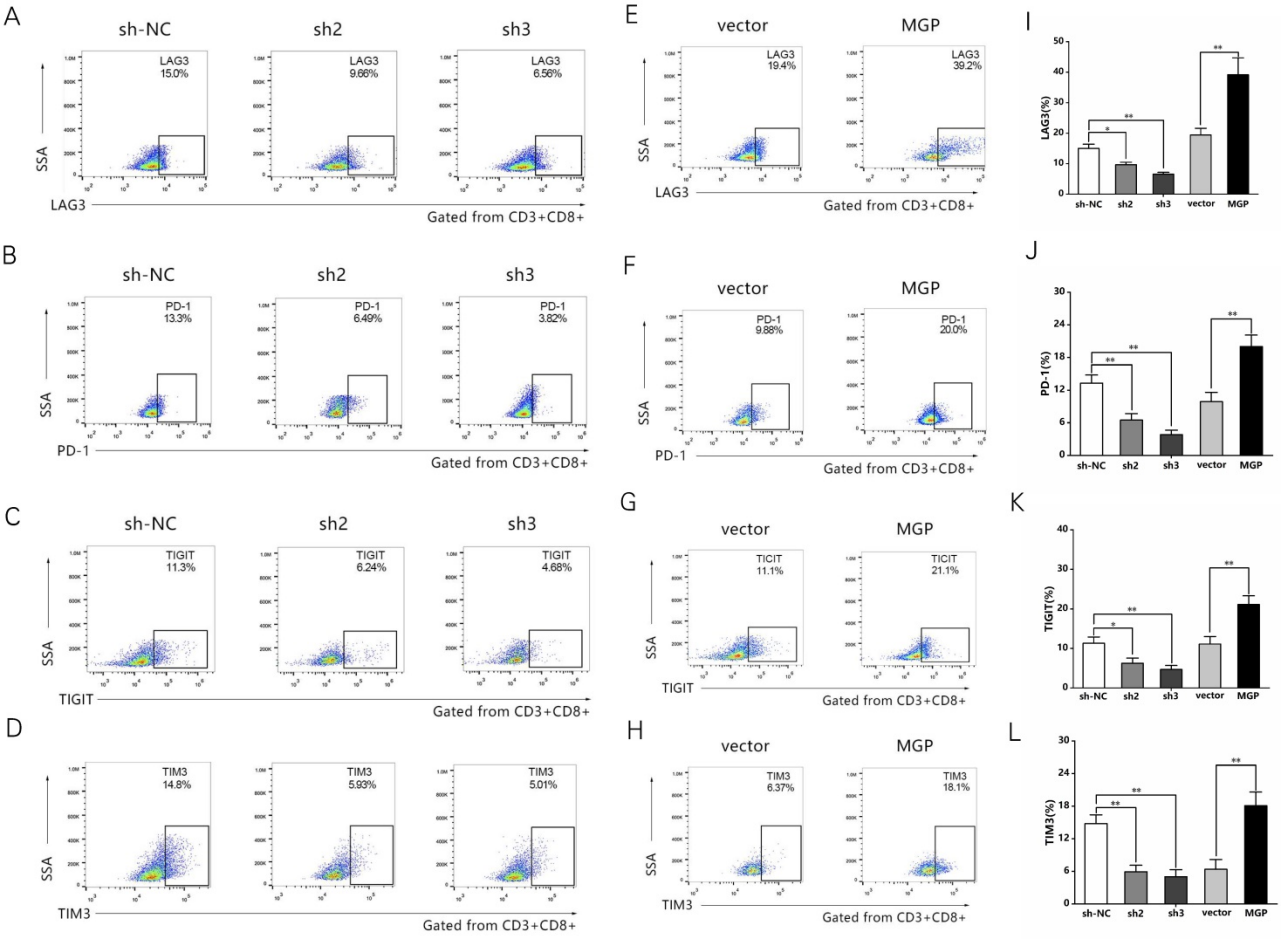
** MGP induced CD8^+^ T cell exhaustion when co-cultured with antigen-specific CD8^+^ T cells isolated from PBMC samples. (A-D)** CRC cells treated with sh2/3-MGP/MGP were co-cultured with antigen-specific CD8^+^ T cells, and the expression of common markers of CD8^+^ T cell exhaustion (LAG3, PD1, TIGIT, and TIM3) was measured by flow cytometry. **(E-H)** CRC cells with MGP overexpression were co-cultured with antigen-specific CD8^+^ T cells, and the expression of common markers of CD8^+^ T cell exhaustion (LAG3, PD1, TIGIT, and TIM3) was measured by flow cytometry. **(I-L)** Statistical analysis of flow cytometry data presented in A-H. *, *P* < 0.05; **, *P* < 0.01.

To examine the effect of MGP on the microenvironment of CRC, the correlation between MGP expression and immune factors was predicted using the TISIDB database. The results indicated a significant positive correlation between MGP expression and the expression of immunoinhibitors (for example, TIGIT, PD1, LAG3, and CTLA4; Figure S3). To further confirm this effect, human CRC cells with knockdown (sh2-MGP and sh3-MGP) or overexpression of MGP were co-cultured with antigen-specific CD8^+^ T cells isolated from PBMC samples. Flow cytometry was performed to measure the expression of immune factors. According to the results, when CRC cells with downregulated expression of MGP were co-cultured with antigen-specific CD8^+^ T cells, the expression of common markers of CD8^+^ T cell exhaustion (for example, LAG3, PD1, TIGIT, and TIM3) was noticeably decreased (Figure 4A-4D, Figure 4I-4L). In contrast, co-culturing of CRC cells overexpressing MGP with antigen-specific CD8^+^ T cells promoted CD8^+^ T cell exhaustion (Figure 4E-4H, Figure 4I-4L).

### MGP upregulated PD-L1 expression in CRC cells

**Figure 5:**
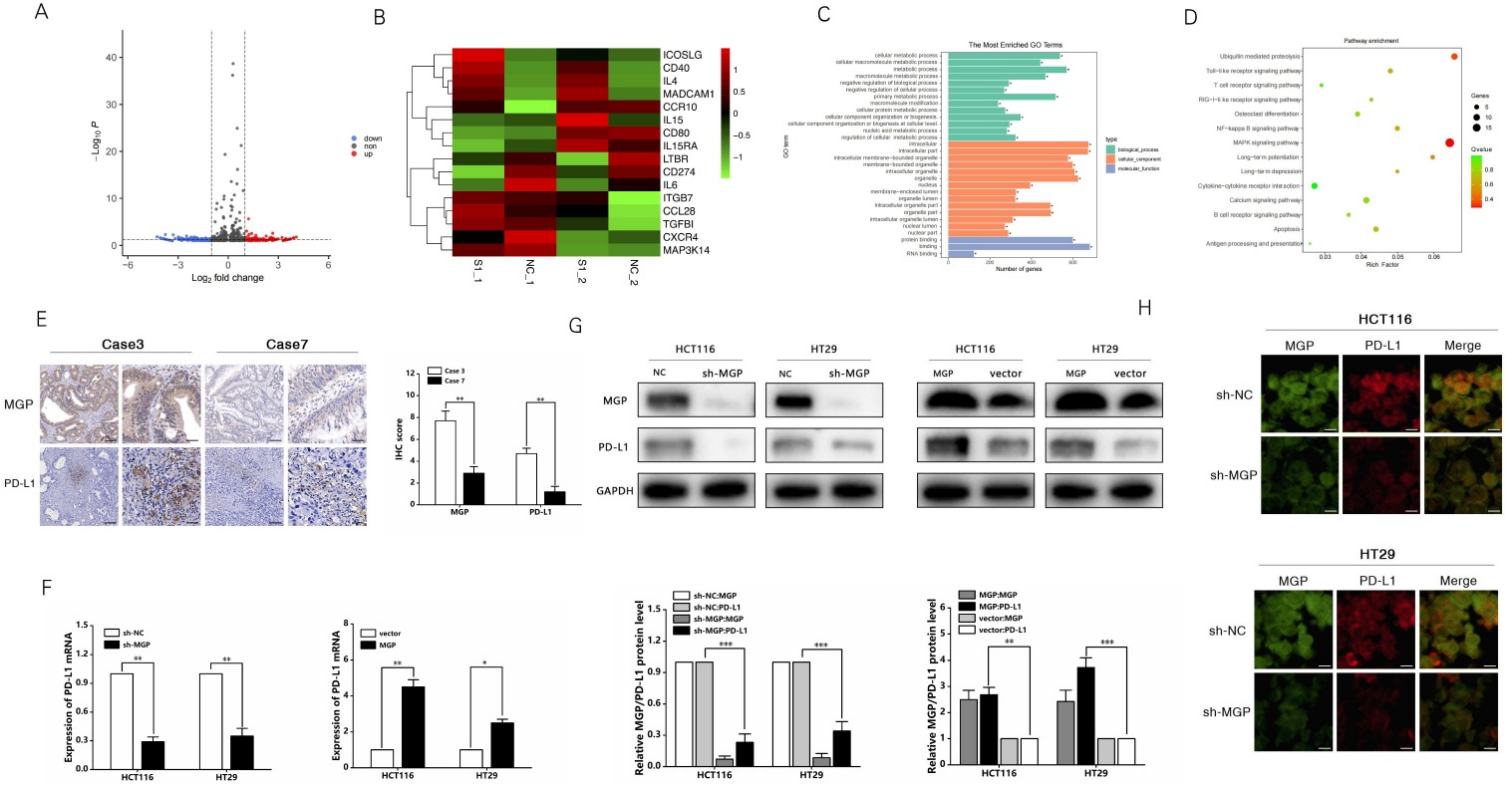
** MGP upregulated PD-L1 expression in CRC. (A)** RNA sequencing was used to assess differentially expressed mRNAs in the sh-MGP and sh-NC groups. In Volcano map, red color indicates higher expression of genes in the sh-MGP group than that in the sh-NC group, and blue represents lower expression. **(B)** Heatmap of differentially expressed mRNAs in the sh-MGP and sh-NC groups. The more intense the red color, the higher the expression. **(C)** GO analysis of mRNAs in CRC cells treated with sh-MGP and sh-NC. **(D)** KEGG pathway analysis of mRNAs in CRC cells treated with sh-MGP and sh-NC. The bigger the bubble, the more is the number of genes. **(E)** Immunohistochemistry was employed to verify the protein expressions of MGP and PD-L1 in CRC tissues of two cases (case 3 with a high MGP expression and case 7 with a low MGP expression). **(F)** The mRNA expression of PD-L1 in CRC cells with MGP knockdown or overexpression was assessed by qRT-PCR. **(G)** The protein expression of MGP and PD-L1 in CRC cells with MGP knockdown or overexpression. The upper panel represents the variation of protein bands, and the lower panel illustrates the protein gray value analysis. **(H)** Immunofluorescence was employed to verify MGP and PD-L1 expression in CRC cells with MGP knockdown. *, *P* < 0.05; **, *P* < 0.01; ***, *P* < 0.001.

To examine the mechanism of inducing CD8^+^ T cell exhaustion by MGP in CRC cells and assess the effects of downstream gene expression of MGP, we performed RNA sequencing (lncRNAs, circRNAs, and mRNAs). Compared with the sh-NC and sh-MGP groups, 381 mRNAs were significantly upregulated and 428 mRNAs were significantly downregulated in the sh-MGP group (Figure 5A). According to the heatmap analysis, immunostimulators (ICOSLG and CD40) were upregulated, whereas immunoinhibitors (IL6 and CD274 [PD-L1]) were downregulated (Figure 5B). Thus PD-L1 expression was significantly downregulated in CRC cells with MGP knockdown (Figure 5B). According to the results of GO analysis, molecular mechanisms such as catalytic activity, binding, and signal transduction activity and biological processes such as cellular polymer metabolism and biogenesis were among the most obviously involved processes (Figure 5C). As revealed by the corresponding KEGG analysis results, differentially expressed genes were largely enriched in the T cell receptor signaling pathway, calcium signaling pathway, and NF-κB pathway (Figure 5D). Furthermore, differentially expressed lncRNAs and circRNAs were analyzed and found to be enriched in immune pathways (Figure S4).

Based on the sequencing results, PD-L1 was used as a target to further identify the mechanism by which MGP affects the development of CRC. The correlation between MGP and PD-L1 expression was predicted using the TISIDB database (Figure S5A). As revealed by immunohistochemistry, high expression of MGP in patients with CRC was associated with an upregulated PD-L1 expression, and *vice versa* (Figure 5E). qRT-PCR and western blotting were performed to compare the variations in PD-L1 mRNA and protein expression after MGP knockdown and overexpression, which revealed that with decrease in MGP expression, PD-L1 mRNA and protein expression also decreased (Figure 5F and 5G). In contrast, overexpression of MGP showed a significant upregulation of PD-L1 (Figure 5F and 5G). Immunofluorescence assay also showed consistent result that PD-L1 expression was reduced at the protein level when MGP was downregulated (Figure 5H). However, a model with downregulated expression of PD-L1 had an insignificant effect on MGP, indicating that PD-L1 was regulated by MGP upstream genes (Figure S5B and S5C).

### MGP promoted calcium influx and activated NF-κB pathway to upregulate PD-L1 expression

**Figure 6:**
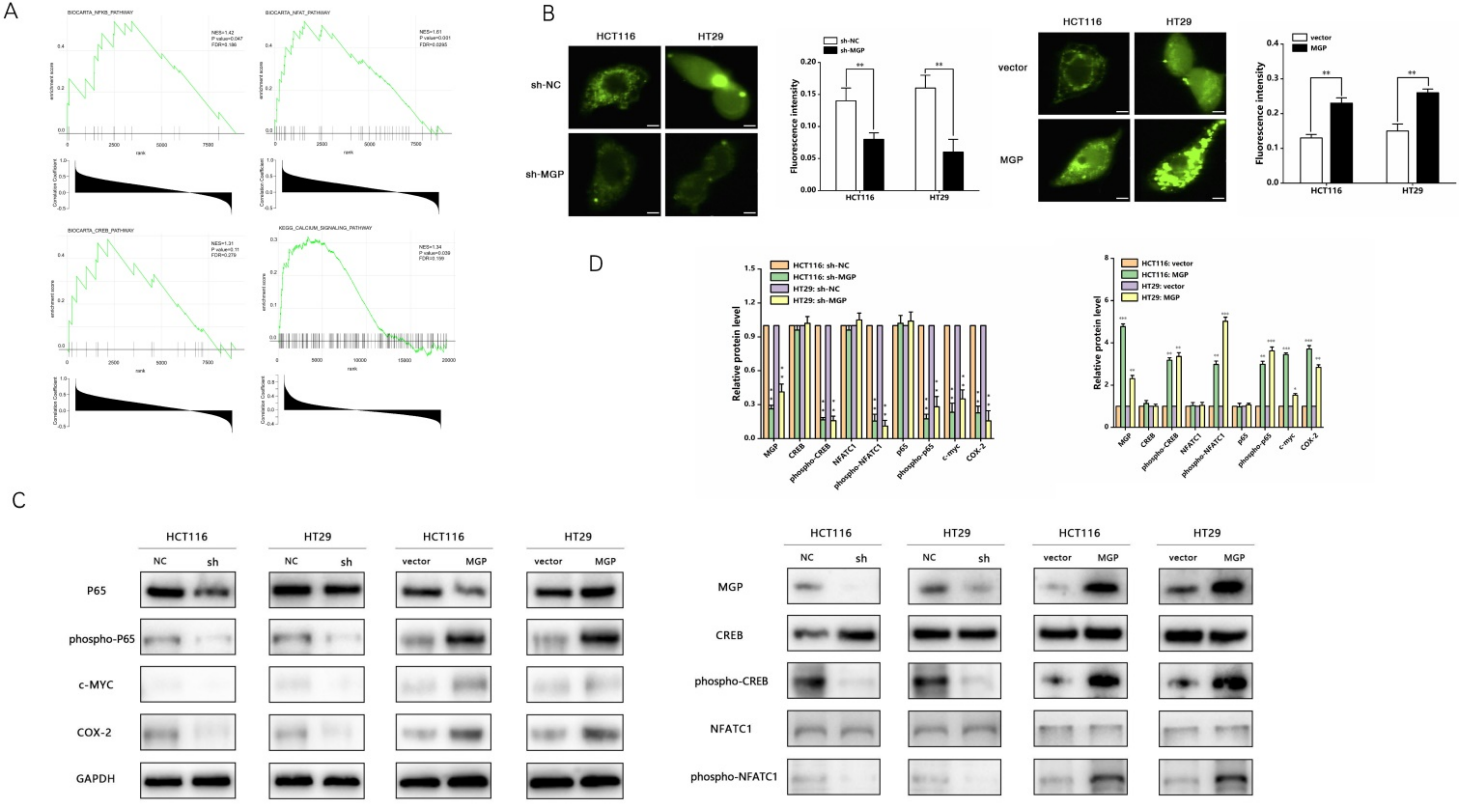
** MGP promoted calcium influx and activated the NF-κB pathway. (A)** GSEA analysis was used to predict the pathways of MGP enrichment; overall, four pathways were selected. Although the *P* value of MGP and CREB pathway enrichment was 0.11, its enrichment was obvious. **(B)** Immunofluorescence assay was performed to detect the calcium influx in CRC cells with MGP knockdown or overexpression. The respective images are accompanied by quantitative calcium ion fluorescence analysis. **(C)** Western blotting revealed the expression of p65, phospho-p65, CREB, phospho-CREB, NFATC1, phospho-NFATC1, c-MYC, and COX-2 in CRC cells with MGP knockdown or overexpression. **(D)** Protein gray value analysis result of C.*, *P* < 0.05; **, *P* < 0.01; ***, *P* < 0.001.

According to GSEA enrichment analysis, there existed a close correlation between MGP and the calcium signaling, NF-κB, NFAT, and CREB pathways (Figure 6A). Therefore, we performed an immunofluorescence assay that indicated a noticeable decrease in the calcium influx after MGP downregulation; moreover, MGP overexpression significantly elevated the calcium influx (Figure 6B). Furthermore, we explored whether MGP can activate the NF-κB pathway and thus affect PD-L1 expression. Interestingly, we found that downregulation of MGP expression inhibited the phosphorylation of p65, CREB, and NFATC1. Furthermore, the protein expression of c-MYC and COX-2 was downregulated when MGP was decreased (Figure 6C and 6D). However, MGP overexpression activated the NF-κB pathway and upregulated the corresponding gene expression (Figure 6C and 6D).

**Figure 7:**
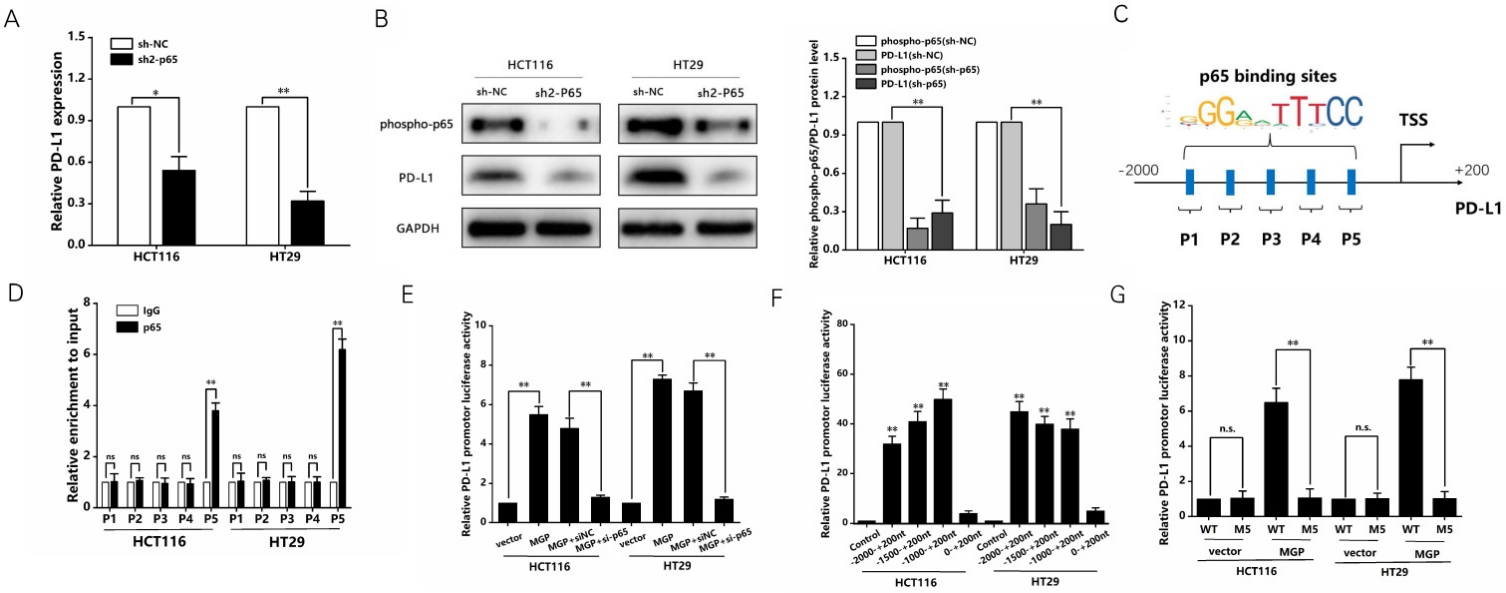
** NF-κB transcription induced the expression of PD-L1 in CRC cells. (A)** The mRNA expression of PD-L1 in CRC cells with p65 knockdown. We used sh2-p65 to downregulate p65. **(B)** The protein expression phosphorpho-p65 and PD-L1 in CRC cells with p65 knockdown. Left panel represents the result of protein banding. Right panel illustrates the result of protein gray value analysis. **(C)** According to the JASPAR database analysis, the promoters of the PD-L1 gene can comprise five p65 binding sites (P1, P2, P3, P4, and P5). **(D)** ChIP assay confirmed that p65 can be directly correlated with the PD-L1 promoters within P5, while it has no obvious significance in other sites (P1, P2, P3, and P4). **(E)** PD-L1 promoter-driven reporter activity was measured under MGP overexpression or MGP plus silencing p65 using si-p65. **(F)** The detection of the five sites tended to narrow down and respectively showed the promoter-driven reporter activity of PD-L1. **(G)** The luciferase reporter gene experiment revealed the result of the mutated (M5) and WT (wild type) sequence affecting the promoter-driven reporter activity of PD-L1 under MGP overexpression.*, *P* < 0.05; **, *P* < 0.01.

Previous studies have reported that the NF-κB pathway is capable of upregulating PD-L1 expression 19. Therefore, we speculated that MGP can upregulate PD-L1 expression by activating the NF-κB pathway, leading to immune escape in CRC. According to the results of qRT-PCR and western blotting, when p65 was downregulated, the mRNA and protein expression of PD-L1 was significantly reduced (Figure 7A and B, Supplementary Figure S5D). As indicated by the hTFtarget database, p65 and PD-L1 occupied the transcriptional regulatory sites (Figure S5E). According to the JASPAR database, PD-L1 gene promoters were predicted to cover five p65 binding sites (Figure 7C). As demonstrated by the ChIP assay, p65 was directly correlated with the PD-L1 promoter within P5 in CRC cells (Figure 7D). Furthermore, MGP overexpression noticeably improved reporter activity driven by the PD-L1 promoter, whereas such an effect in CRC cells was offset by MGP overexpression and silencing of p65 (Figure 7E). The detection of the five sites tended to narrow down, indicating that the PD-L1 promoter-driven reporter activity was progressively improved. However, when the sites were far away from P5, the promoter-driven reporter activity of PD-L1 was low (Figure 7F). As revealed by the luciferase reporter gene experiment, after MGP was overexpressed, the mutated sequence did not affect the PD-L1 promoter-driven reporter activity of, while the wild type had a significant effect (Figure 7G). The ChIP data from GSE131710 showed that p65 had a significant peak in the upstream of PD-L1 and the motif is TGGACTTTCC, suggesting that there was an obvious binding site between p65 and PD-L1 (Figure S6).

**Figure 8:**
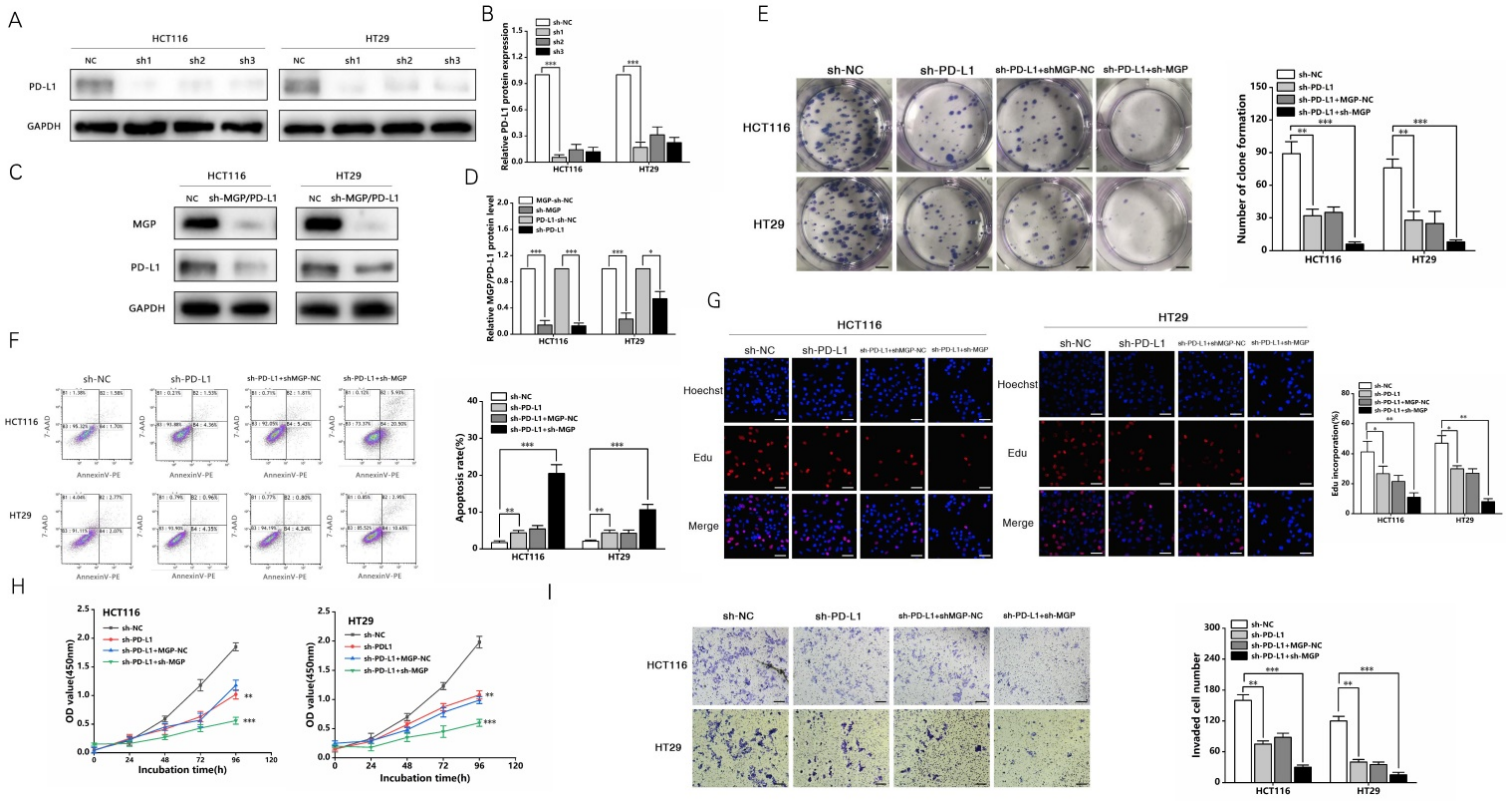
** sh-MGP and sh-PD-L1 inhibited the proliferation and invasion of CRC cells. (A)** Three shRNA against PD-L1 (sh-PD-L1) were designed to silence PD-L1 in CRC cells, and validated by western blotting. **(B)** The gray value analysis result of A. **(C)** sh3-MGP and sh3-PD-L1 was validated by western blotting in CRC cells. **(D)** The gray value analysis result of C. **(E)** Colony formation assay was performed to assess cell proliferation in four groups. **(F)** Flow cytometry was used to assess cell apoptosis in four groups. **(G)** EdU assay of CRC cells transfected with sh-PD-L1/sh-PD-L1+sh-MGP was performed to assess cell proliferation. **(H)** The growth curves of cells after transfection with sh-PD-L1/sh-PD-L1+sh-MGP using CCK-8 assay. **(I)** Transwell invasion assay was used to assess cell invasion in four groups. *, *P* < 0.05; **, *P* < 0.01; ***, *P* < 0.001.

**Figure 9:**
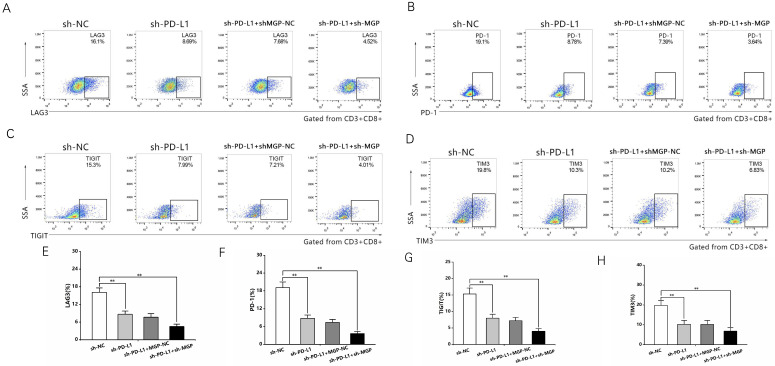
** sh-MGP and sh-PD-L1 reduced CD8^+^ T cell exhaustion when co-cultured with antigen-specific CD8^+^ T cells isolated from PBMC samples. (A-D)** CRC cells with sh-PD-L1/sh-MGP+sh-PD-L1 were co-cultured with antigen-specific CD8^+^ T cells, and the expression of common markers of CD8^+^ T cell exhaustion (TIGIT, LAG3, PD1, and TIM3) was measured by flow cytometry. **(E-H)** Statistical analysis of flow cytometry data presented in A-D. **, *P* < 0.01.

In addition, a PD-L1 protein reduction model was successfully established (Figure 8A and 8B). We used sh3-MGP and sh3-PD-L1 to verify the knockdown levels *via* qRT-PCR and western blotting (Figure 8C and 8D, Figure S7). To further verify the correlation between MGP and PD-L1, PD-L1 and MGP expression was simultaneously knocked down, and it was found that this could significantly inhibit the proliferation and invasion of cancer cells and facilitate the apoptosis of cancer cells, compared with the unilateral knockdown of PD-L1 (Figure 8E-8I). When sh-PD-L1+sh-MGP CRC cells were co-cultured with antigen-specific CD8^+^ T cells, the expression of common markers of CD8^+^ T cells exhaustion was downregulated significantly (Figure 9).

### Inhibition of MGP reduced liver metastasis and increased the efficiency of αPD1 treatment in CRC

**Figure 10:**
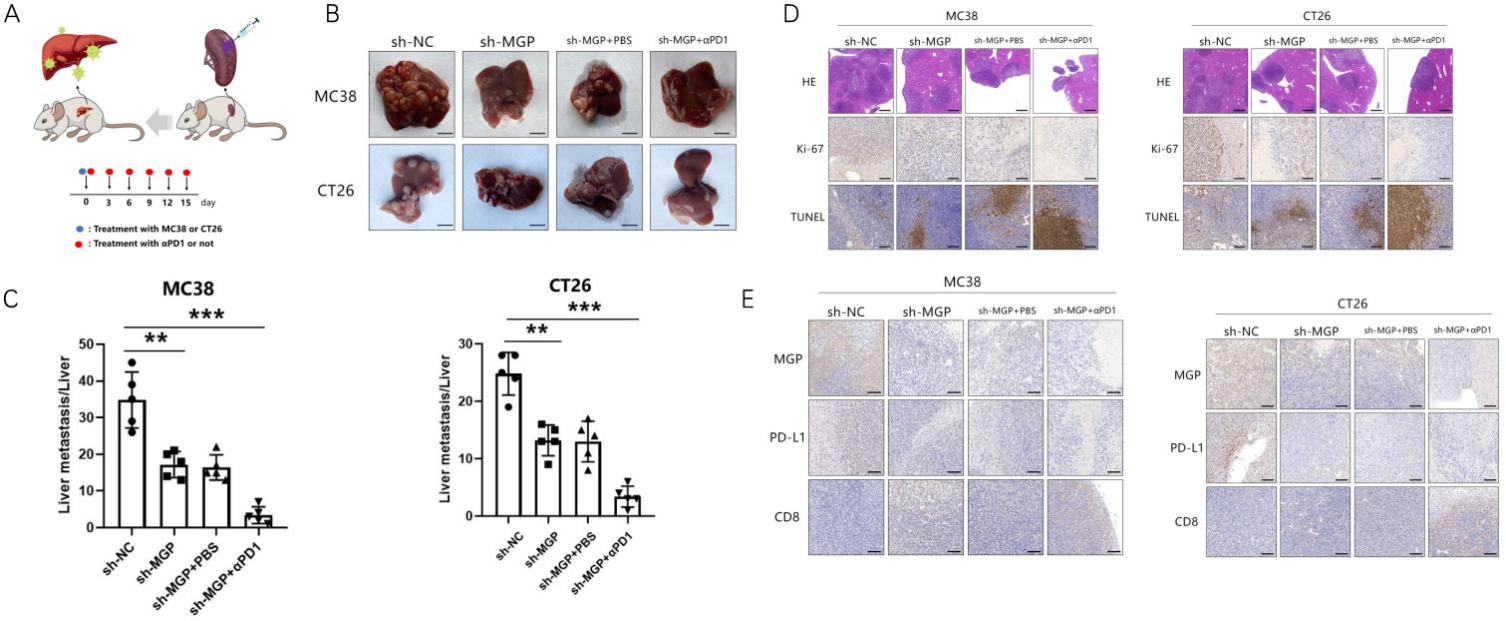
** Inhibition of MGP reduced liver metastasis and increased the efficacy of αPD1 treatment against CRC. (A)** Procedure for establishing liver metastasis of CRC. PD1 antibody was injected intraperitoneally on the day of spleen injection and every three days thereafter. **(B)** Representative images of liver metastases in the respective groups (sh-NC, sh-MGP, sh-MGP+PBS, and sh-MGP+αPD1). **(C)** Analysis of liver metastases in the respective groups. **(D)** Liver metastases in four groups were confirmed by HE staining. Immunohistochemistry result of Ki-67 and TUNEL expression in the respective groups. **(E)** Immunohistochemistry results of MGP, PD-L1, and CD8 expression in the respective groups. **, *P* < 0.01; ***, *P* < 0.001.

**Figure 11:**
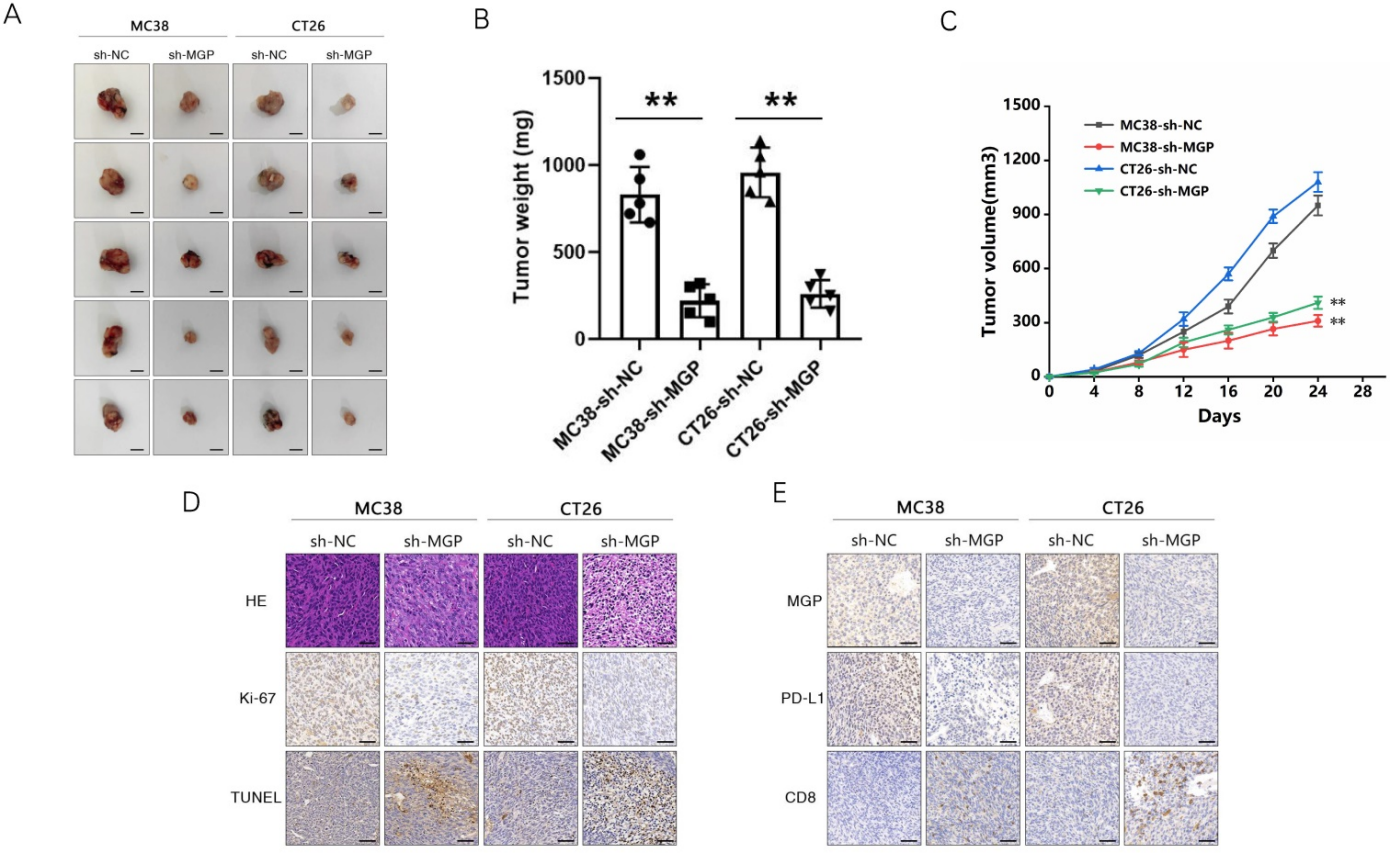
** Inhibition of MGP inhibited subcutaneous tumor of CRC cells. (A)** Representative images of subcutaneous tumors in the respective groups (sh-NC and sh-MGP). **(B-C)** The volume (B) and weight (C) statistics of subcutaneous tumors in the respective groups (sh-NC and sh-MGP). **(D)** The tumors were confirmed by HE staining. Immunohistochemistry result of Ki-67 and TUNEL expression in the respective groups. **(E)** Immunohistochemistry result of MGP, PD-L1, and CD8 expression in the respective groups.**, *P* < 0.01.

To explore the correlation between MGP and the growth and metastasis of CRC *in vivo*, the sh-MGP and sh-NC groups were injected MC38 and CT26 cells into the spleen of C57BL/6 and BALB/C mice, respectively, followed by αPD1 injection to assess the hepatic metastatic capacity of these cells (Figure 10A). The downregulated expression of MGP in the sh-MGP group significantly inhibited liver metastasis of CRC, compared with the sh-NC group. Moreover, when αPD1 was used simultaneously, the inhibition rate of liver metastasis in the sh-MGP group was significantly increased compared with that of the PBS group (Figure 10B and 10C). Hematoxylin and eosin (HE) staining was performed to confirm liver metastasis. According to the immunohistochemistry results, Ki-67 expression was significantly decreased, and apoptosis was noticeably facilitated when αPD1 was used simultaneously (Figure 10D); additionally, MGP and PD-L1 expression in the liver tissues was downregulated and CD8 infiltration was elevated (Figure 10E). In addition, the effect of MGP was assessed using a subcutaneous tumor-bearing model in C57/B6 and BALB/C mice; the volume and weight of the tumor decreased significantly after MGP knockdown (Figure 11A-11C). Immunohistochemistry results showed that Ki-67, PD-L1, and MGP expression was noticeably decreased, and apoptosis and CD8 infiltration was significantly increased (Figure 11D-11E). Collectively, this study revealed that inhibition of MGP could reduce liver metastasis and improve the efficiency of αPD1 treatment in CRC.

**Figure 12:**
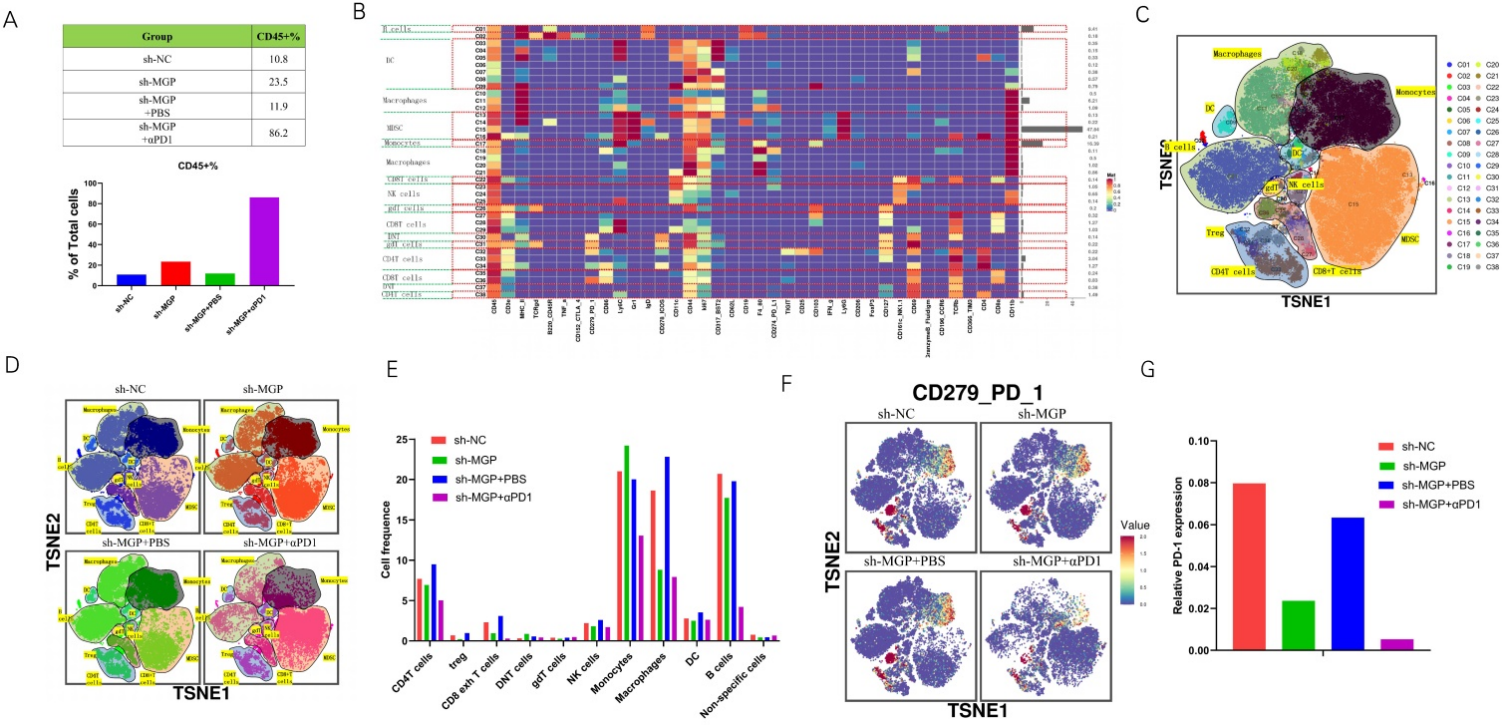
** Mass cytometry reflected the immune microenvironment of CRC liver metastasis after sh-MGP/sh-MGP+αPD1 treatment. (A)** We cycled the selected single, live, and intact CD45^+^ immune cells from the liver cancer tissues of the respective groups of and calculated the number of CD45^+^ cells in each group. **(B)** There were 38 cell clusters in total, which were defined in the respective groups. **(C)** TSNE plot showing distribution of 38 cell clusters. **(D)** TSNE diagram showing distribution of cell clusters in the respective samples. **(E)** The histogram showing the number of the respective cell clusters in different groups by mass cytometry. **(F)** TSNE plot showing distribution of PD1 in four groups. **(G)** The histogram showing the number of PD1^+^ cell clusters in different groups.

To further assess the variations in the immune microenvironment of the liver cancer tissues in the sh-MGP and sh-NC groups, we used mass cytometry to measure the expression of the respective immune cell clusters. We cycled single, live, and intact CD45^+^ immune cells from the selected cells in the respective liver tissues (Figure S8), and found that compared with the sh-NC group, the number of CD45^+^ cells in the sh-MGP group increased significantly, which demonstrated that the knockdown of MGP enhanced the immune invasion of the tumor. After αPD1 treatment, the number of CD45^+^ cells increased further (Figure 12A). All samples showed clustering and subgroup annotation of CD45*^+^* immune cells. There were 38 cell clusters in total, and the respective cell clusters were defined based on the specific markers of the respective cell types (Figure 12B and 12C, Figure S9). According to the results, immunosuppressive cell population, including CD8^+^ exhausted T cells, regulatory T cells (Tregs), and macrophages decreased after MGP downregulation, and the expression of these cell populations was further downregulated when αPD1 was used simultaneously (Figure 12D and 12E). In addition, we assessed the overall expression of PD1 in the immune microenvironment. According to the results, PD1 expression was noticeably downregulated after MGP knockdown and further decreased after αPD1 treatment (Figure 12F and 12G). These results demonstrate that MGP knockdown in CRC cancer cells could lead to a decrease in PD-L1 expression on the surface of cancer cells and decrease the number of immunosuppressive cells. Therefore, the combination of MGP knockdown and αPD1 is beneficial for synergistic resistance against CRC liver metastasis.

## Discussion

A recent study reported that MGP was overexpressed in various tumors (for example, breast cancer 20, ovarian cancer 21, and gastric cancer 22), which demonstrated that it may be related to the progression and invasion of tumors. In this study, we revealed that MGP expression was higher in CRC tissues than that in paracancerous tissues. Kaplan-Meier curves indicated that patients with CRC and higher MGP expression had shorter overall survival. Helena *et al.* 23 observed that MGP was overexpressed in CRC tumor mucosa at the mRNA level, in contrast with the surrounding normal mucosa; moreover, higher MGP mRNA expression was associated with a poorer prognosis and an overall survival of less than five years. Our study employed the latest technique of single-cell RNA sequencing to further verify MGP expression in CRC cancer cell clusters at the single-cell level, thereby making our conclusion more credible. MGP has been suggested as a prognostic factor in CRC cases, thereby emphasizing the significance of MGP as a biomarker marker.

To the best of our knowledge, this is the first study to report that MGP can facilitate CRC liver metastasis. Previous studies have reported the role of MGP in CRC, but the studies did not involve metastasis. For instance, Li *et al.* 24 reported that MGP promoted the growth and proliferation of colon cancer cells. Huang *et al.* 25 reported that suppression of MGP or oxaliplatin treatment alone significantly inhibited tumor growth in a CRC mouse model. The *in vitro* experiments in the present study revealed that MGP could facilitate the proliferation and invasion of CRC cells. According to the *in vivo* results, the downregulated expression of MGP significantly inhibited liver metastasis of CRC, compared with the sh-NC group. Our study results add new insights into the metastatic function of MGP in cancer.

This study highlighted the significance of MGP in promoting CRC liver metastasis in the tumor microenvironment, in contrast with the existing studies that were limited to cancer cells only and could not effectively demonstrate the function of MGP in cancer tissues. Over the past two decades, most cancer immunotherapies were focused on enhancing T lymphocyte-mediated adaptive immunity against tumors. However, treatment strategies involving IL-2 26 or autologous T lymphocyte infusion 27 led to mild or no success and high toxicity. Over the last five years, immunotherapy approaches employing single immune checkpoint blockade (ICB) agents have achieved dramatic clinical efficacy in several types of cancers 28. ICB-based cancer immunotherapies, particularly involving the T cell immune checkpoint inhibitors anti-CTLA-4 and anti-PD1 have provided consistent clinical benefits in various cancer cases 29. The use of ICB-based monotherapy (anti-CTLA-4, anti-PD1, or anti-PD-L1) has been severely impaired, although there have been exciting and promising clinical responses in various malignancies. According to the clinical data, most patients treated with ICB achieved an incomplete response or failed to achieve higher objective responses 30. This study proved that MGP could facilitate calcium influx and activate the NF-κB pathway to upregulate PD-L1 expression. Li *et al.* 24 demonstrated that MGP promoted colon cancer cell proliferation by activating the NF-κB pathway through upregulation of the calcium signaling pathway, and the potential correlation between MGP and the tumor microenvironment was improved by using PD-L1 as an intermediate vector. We observed that simultaneous knockdown of PD-L1 and MGP significantly inhibited the proliferation and invasion of cancer cells and promoted the apoptosis of cancer cells, compared with the unilateral knockdown of PD-L1. When sh-PD-L1+sh-MGP CRC cells were co-cultured with antigen-specific CD8^+^ T cells, the expression of common markers of CD8^+^ T cell exhaustion was noticeably decreased. Furthermore, according to *in vivo* experiments, inhibition of MGP significantly lowered the rate of liver metastasis of CRC and enhanced the efficacy of αPD1 in CRC therapy. To further assess the variations in the immune microenvironment of the liver cancer tissues in the sh-MGP and sh-NC groups, we used mass cytometry to measure the expression of the respective immune cell clusters. We observed that immunosuppressive cell population (for example, CD8^+^ exhausted T cells, Tregs, and macrophages) was reduced after MGP downregulation, and the expression of these cell populations was further downregulated when αPD1 was used simultaneously. These results proved that the combination of MGP knockdown and αPD1 could synergistically resist CRC liver metastasis.

In conclusion, this study revealed that MGP could facilitate CD8^+^ T cell exhaustion by activating the NF-κB pathway, thereby leading to liver metastasis of CRC. Moreover, the combination of MGP knockdown and αPD1 might have contributed to synergistic resistance against CRC liver metastasis. A novel strategy and method was developed to improve the therapeutic efficacy of αPD1, exploring new prospects for treating liver metastasis of CRC.

## Supplementary Material

Supplementary figures and tables.
