# Supplementary material for: MGP promotes CD8+ T cell exhaustion by activating the NF-κB pathway leading to liver metastasis of colorectal cancer
Source: Int J Biol Sci. 2022 Mar 6;18(6):2345–61. doi: 10.7150/ijbs.70137 (PMC8990480; doi:10.7150/ijbs.70137)
Supplement: Supplementary file 1 — Supplementary figures and tables. [file ijbsv18p2345s1.pdf]

Supplementary figure legends:

Figure S1: MGP expression in metastatic liver tissues and normal liver tissues of mouse model. The left panel represents the protein expression of MGP in metastatic liver tissues and normal liver tissues of mouse model using western blotting. The right panel shows the result of the protein gray value analysis. GAPDH was used as the loading control. *, $P < 0.05$; **, $P < 0.01$.

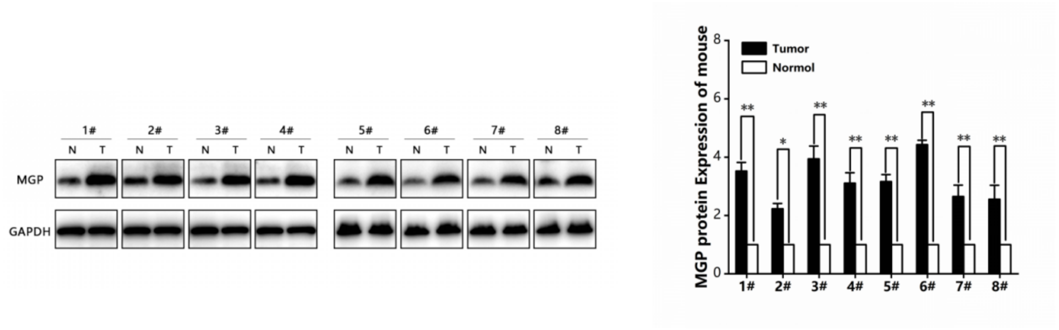

Figure S2: Validation of knockdown and overexpression of MGP in CRC cells. (A) qRT-PCR was used to measure the expression of three MGP shRNA in CRC cells (HCT116 and HT29). **(B)** qRT-PCR was used to measure the expression of MGP in CRC cells overexpressing MGP (HCT116 and HT29). **, $P < 0.01$; ***, $P < 0.001$.

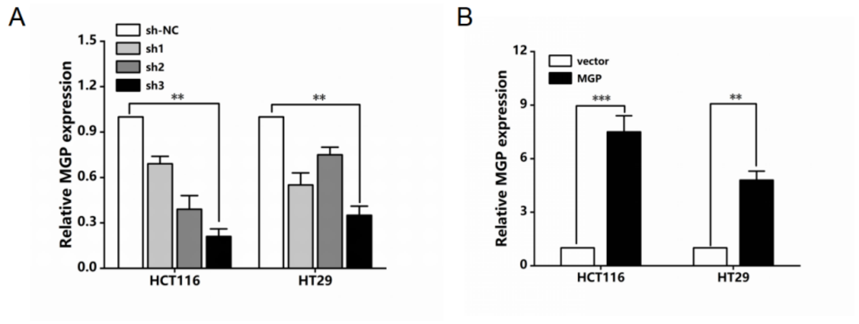

Figure S3: Correlation between MGP expression and immunosuppressive cell expression. The TISIDB database prediction indicated a significant correlation between MGP expression and the expression of immunoinhibitors, including TIGIT (A), PD1 (B), LAG3 (C), and CTLA4 (D).

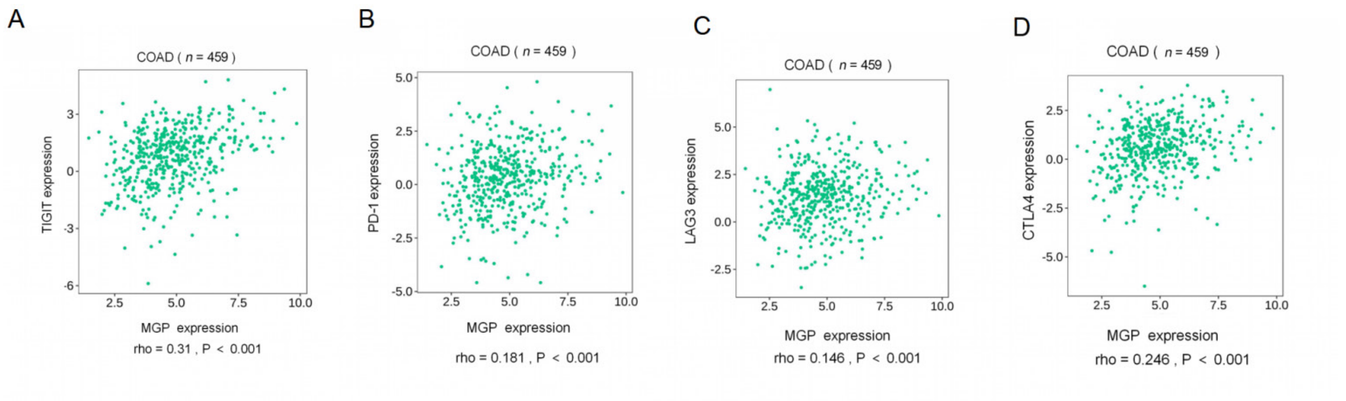

Figure S4: Differentially expressed lncRNAs and circRNAs in the sh-MGP and sh-NC groups. (A) RNA sequencing was employed to assess differentially expressed lncRNAs in the sh-MGP and sh-NC groups. In volcano map, red color represents higher expression of genes in the sh-MGP group than that in the sh-NC group, and green color represents lower expression. **(B)** GO analysis of lncRNAs in CRC cells treated with sh-MGP and sh-NC. **(C)** KEGG pathway analysis of lncRNAs in CRC cells treated with sh-MGP and sh-NC. **(D)** RNA sequencing was employed to assess differentially expressed circRNAs in the sh-MGP and sh-NC groups. Volcano map was presented. **(E)** GO analysis of circRNAs in CRC cells treated with sh-MGP and sh-NC. **(F)** KEGG pathway analysis of circRNAs in CRC cells treated with sh-MGP and sh-NC.

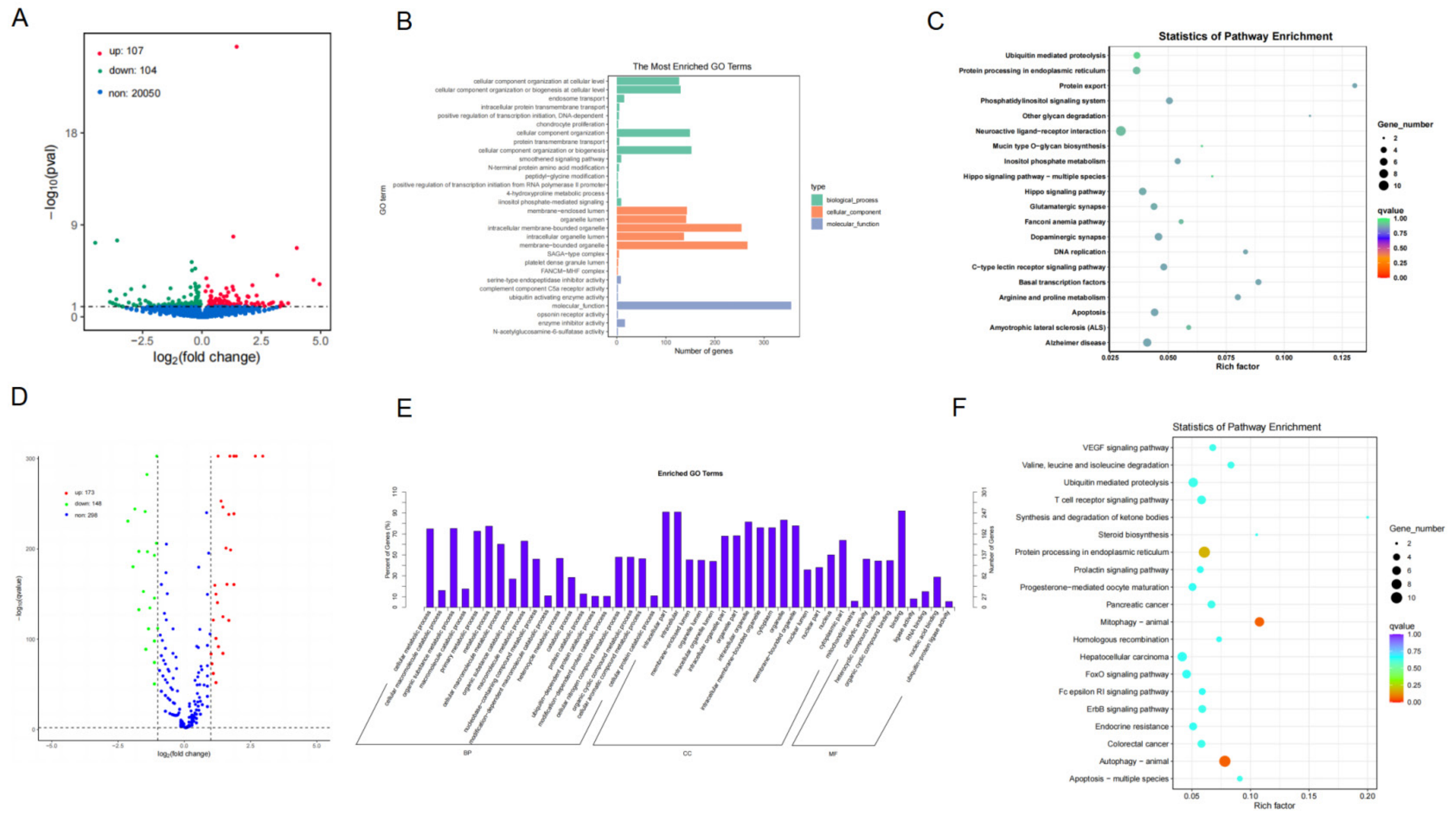

Figure S5: Relationship among MGP, PD-L1, and p65 expression.(A) The TISIDB database predication indicated a significant correlation between the expression of PD-L1 and MGP. **(B)** Three shRNAs (sh1, sh2, and sh3) were designed to silence PD-L1 in CRC cells (HCT116 and HT29), and validated by qRT-PCR. sh1-PD-L1 showed the best knock down effect and was used for subsequent verification.**(C)** qRT-PCR was used to measure MGP expression in CRC cells with PD-L1 knockdown. **(D)** qRT-PCR was used to measure the expression of three shRNA against p65 in CRC cells. **(E)** The hTFtarget database shows that p65 and PD-L1 have transcriptional regulatory sites. **, $P < 0.01$; ***, $P < 0.001$.

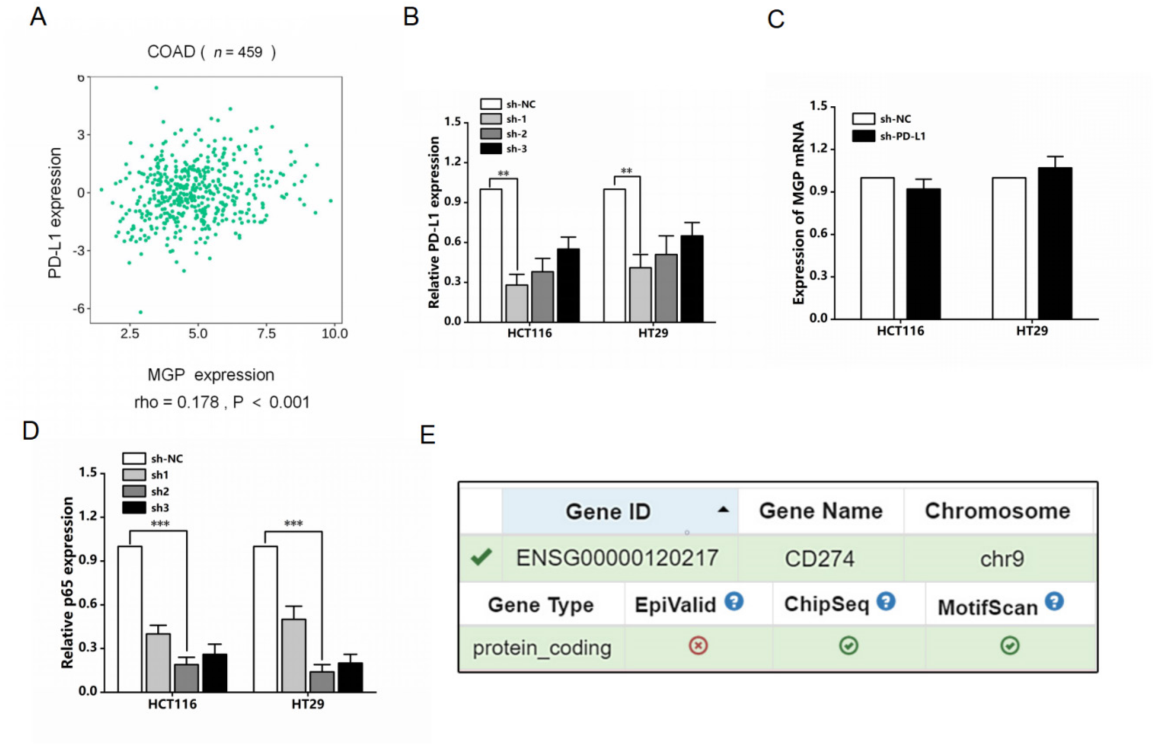

Figure S6: The ChIP data from GSE131710 showed that p65 had a significant peak in the upstream of PD-L1 and the motif is TGGACTTTCC.

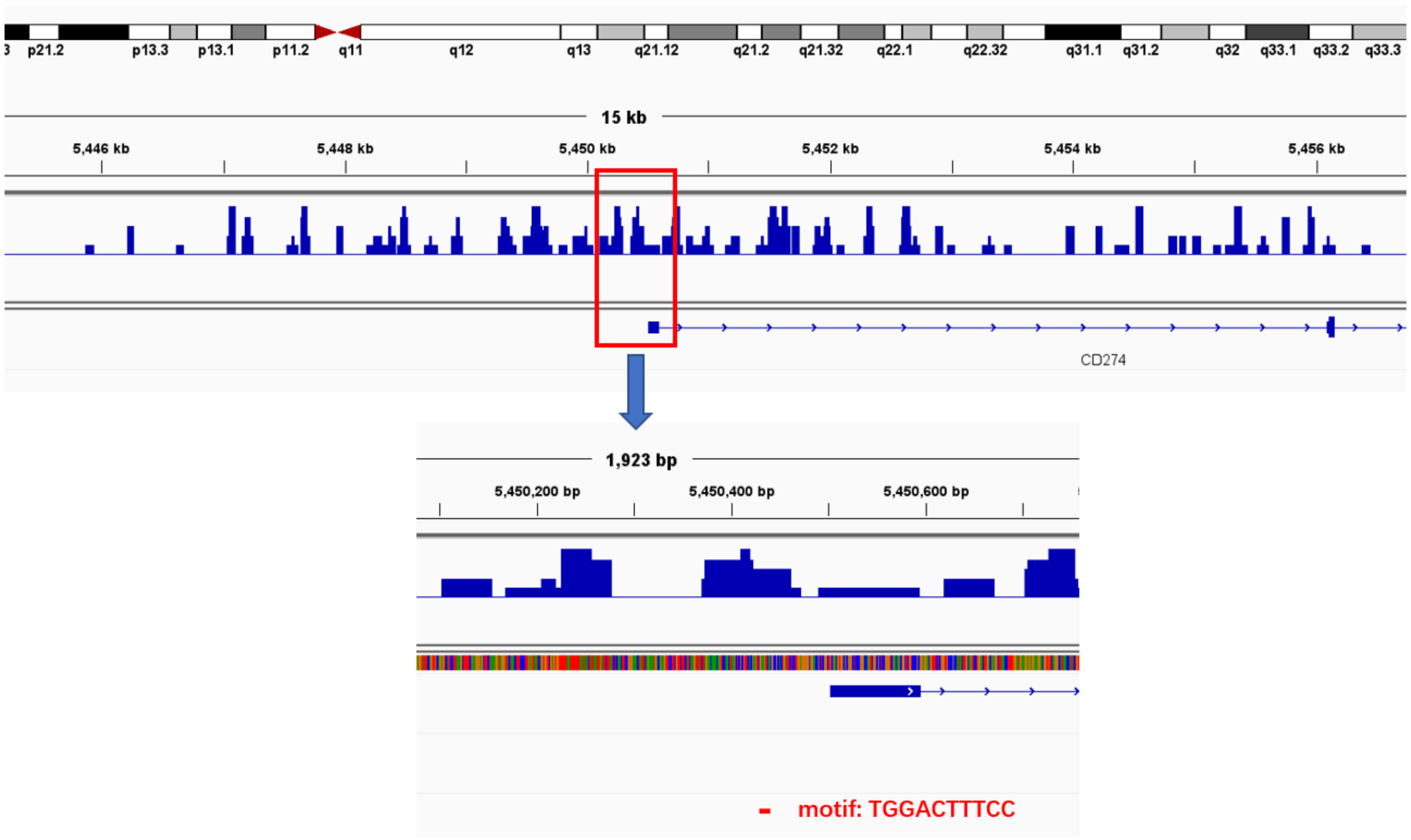

Figure S7: We used sh3-MGP and sh3-PD-L1 to verify the knockdown levels *via* qRT-PCR.

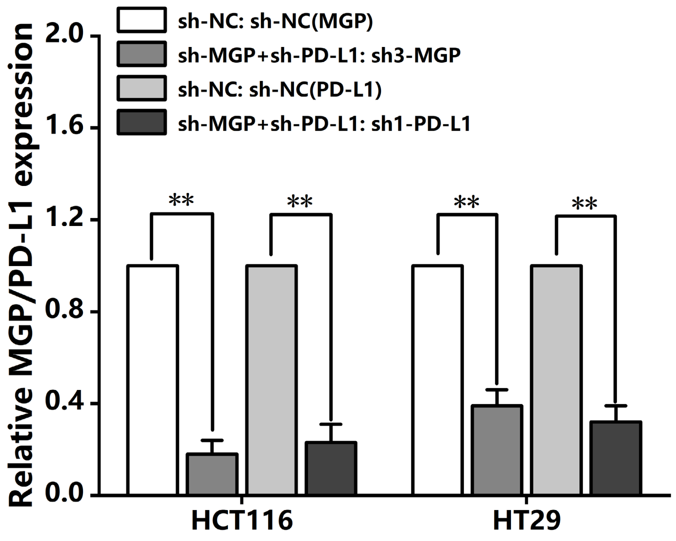

Figure S8: CyTOF staining steps. CyTOF does not have fluorescence flow cytometry FSC and SSC parameters. It can insert DNA only through 193Ir metal and identify cells based on DNA content. We cycled the selected single, live, and intact CD45+ immune cells from the liver tissues of the respective groups.191Ir,193Ir,89Y,194Pt are all metal labels.

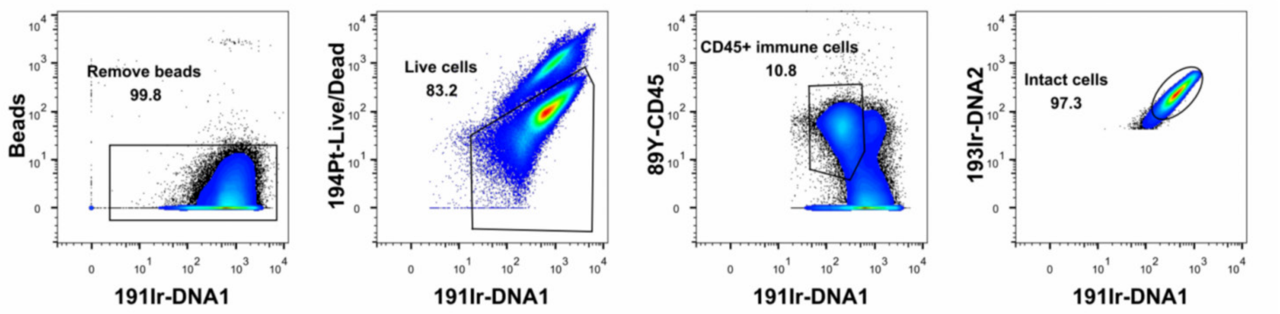

Figure S9: The expression of cell clustering maker genes measured by mass cytometry and presented in the form of TSNE plot.

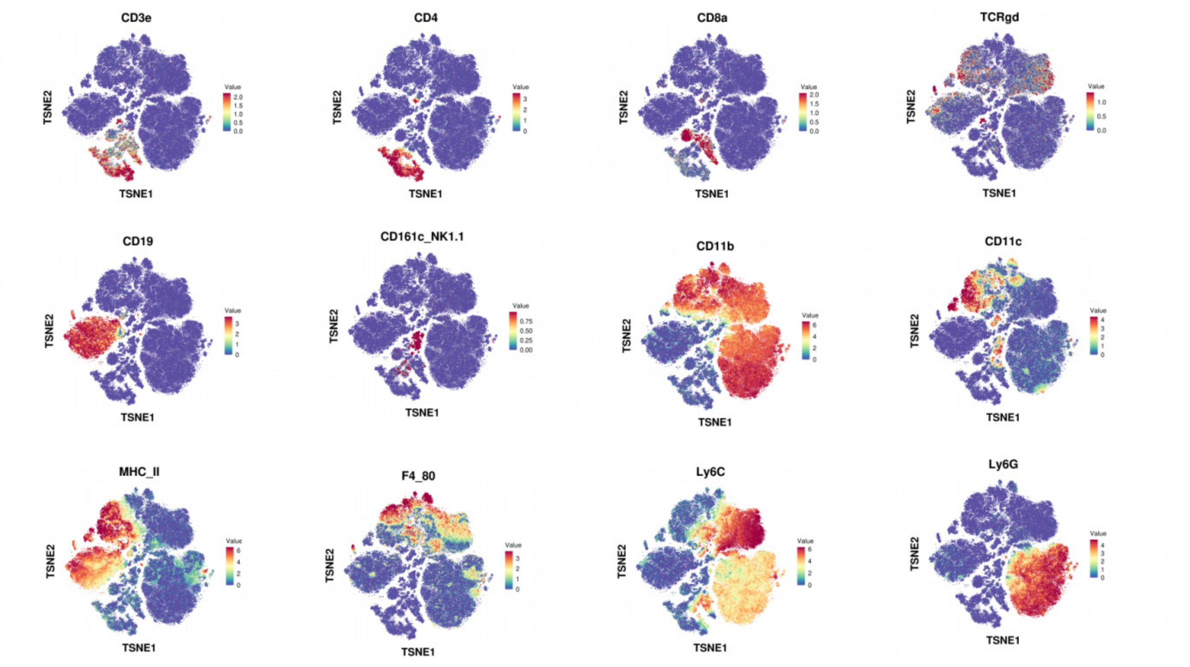

Table S1. **Primer sequences, shRNAs, and siRNAs used in this study.**

Primer/shRNA/siRNA		Sequence (5'-3')
	Forward primer	TCCGAGAACGCTCTAAGCCT
human-MGP	Reverse primer	GCAAAGTCTGTAGTCATCACAGG
	Forward primer	TGGCATTTGCTGAACGCATTT
human-PD-L1	Reverse primer	TGCAGCCAGGTCTAATTGTTTT
	Forward primer	ACCCAGAAGACTGTGGATGG
human-GAPDH	Reverse primer	TCAGCTCAGGGATGACCTTG
	Forward primer	GGCAACCCTGTGCTACGAAT
mouse-MGP	Reverse primer	CCTGGACTCTCTTTTGGGCTTTA
	Forward primer	AGGCCGGTGCTGAGTATGTC
mouse-GAPDH	Reverse primer	TGCCTGCTTCACCACCTTCT
	sh1	GCCTTAGCGGTAGTAACTTTG
	sh2	GCCTGTGATGACTACAGACTT
human-MGP-shRNA	sh3	GCCTATAATCGCTACTTCAGG
	sh1	sh1: GGATCCAGTCACCTCTGAACA
	sh2	sh2: GCCGAAGTCATCTGGACAAGC
human-PD-L1-shRNA	sh3	sh3: GCAGTGACCATCAAGTCCTG
	sh1	sh1:GCCAAATATTAGCGCGAAGAA
	sh2	sh2:AGCCAAATATTAGCGCGAAGA
mouse-MGP-shRNA	sh3	sh3:AGCGCGAAGAAACAGTCATTT

Control shRNA	sh-NC	TTCTCCGAACGTGTCACGTT
	si1 (sense)	CCCACGAGCUUGUAGGAAATT
	si1 (antisense)	UUUCCUACAAGCUCGUGGGTT
	si2 (sense)	GGAGAAACGUAAAAGGACATT
	si2 (antisense)	UGUCCUUUUACGUUUCUCCTT
	si3 (sense)	GGCGAGAGGAGCACAGAUATT
human-p65-siRNA	si3 (antisense)	UAUCUGUGCUCCUCUCGCCTT
	siNC (sense)	UUCUCCGAACGUGUCACGUTT
Control siRNA	siNC (antisense)	ACGUGACACGUUCGGAGAATT
	sh1	CACCGGAGCACAGATACCACCAAGATCAAGAGTCTTGGTGGTATCTGTGCTCC
human-p65-shRNA	sh2	CACCGGGATGAGATCTTCCTACTGTTCAAGAGACAGTAGGAAGATCTCATCCC
	sh3	CACCGGACATATGAGACCTTCAAGATCAAGAGTCTTGAAGGTCTCATATGTCC
	P1-Forward primer	TGGCTGAAGGGTAGAAACAGGT
	P1-Reverse primer	CTCCTAGATGGCCTGGATGATC
	P2-Forward primer	GCCAGGATTAAATCATATCCTCCTAG
	P2-Reverse primer	TGAATGGCTGAAGGGTAGAAACAG
	P3-Forward primer	AATAGGGTTTGGGCCCAGC
	P3-Reverse primer	CATTAAATGAAAATATCAGAGGGCATTGC
	P4-Forward primer	TCTCTTTGGCCCCAATAAAATTGC
	P4-Reverse primer	GCCACATAATGTCTATATTTTCCTAGAGGTC
	P5-Forward primer	GGGAAGCTGCGCAGAACTG
ChIP-qPCR for MGP	P5-Reverse primer	GGTGAAAATCTCATTTACAAGAAAACTGGAC

Table S2. Clinical characteristics of 57 patients with CRC

Characteristic		Total (cases [%])	MGP expression (cases [%]) Low (*n* = 33)	High (*n* = 24)	*P* value
**Age**					0.247
	<65 years	22 (38.6)	11 (19.3)	11 (19.3)	
	≥65 years	35 (61.4)	22 (38.6)	13 (22.8)	
**Gender**					0.166
	Male	35 (61.4)	18 (31.6)	17 (29.8)	
	Female	22 (38.6)	15 (26.3)	7(12.3)	
**Tumor size**					0.352
	<5 cm	29 (50.9)	18 (31.6)	11 (19.3)	
	≥5 cm	28 (49.1)	15 (26.3)	13 (22.8)	
**Differentiation**					0.585
	High-middle	10 (17.5)	6 (10.5)	4 (7.0)	
	Low	47 (82.5)	27 (47.4)	20 (35.1)	
**TNM stage**					<0.001
	I-II	53 (93.0)	32 (56.1)	21 (36.8)	
	III-IV	4 (7.0)	1 (1.8)	3 (5.3)	
**Lymph node metastasis**					<0.001
	Absent	53 (93.0)	32 (56.1)	21(36.8)	
	Present	4 (7.0)	1 (1.8)	3 (5.3)	
